# Mathematical model of dry stack structural elements with geometric imperfections under a cyclic bending moment

**DOI:** 10.1038/s41598-024-61784-y

**Published:** 2024-05-16

**Authors:** Mateusz Smolana, Krzysztof Gromysz

**Affiliations:** Department of Building Structures, Faculty of Civil Engineering, Silesian University of Technology, 44-100, Gliwice, Poland

**Keywords:** Civil engineering, Mechanical engineering

## Abstract

Dry stack structural elements are characterized by nonlinear stiffness that arises from geometric imperfections of their components and the absence of any bonding between them. Moreover, such elements dissipate energy under cyclic loading because of their internal structure. The authors considered dry stack structural elements loaded with a bending moment to propose a relatively simple mathematical model of dry stacks composed of only three elements. The model consists of a linear spring, a nonlinear spring, and a spring with hysteresis in series. In this model, the first element describes the idealized properties of a dry stack element, while the second and third elements correspond to the influence of geometric imperfections and the behaviour of dry joints. Furthermore, the authors described a procedure for determining the parameters of the model based on test results. The proposed solution was verified via experimental studies of temporary support structures consisting of a stack of cuboid elements and a hydraulic jack typically used in the process of building rectification. This study showed that the proposed model adequately describes both the nonlinearity and the energy dissipation under a cyclic bending moment.

## Introduction

Dry stack structural elements provide interesting solutions when utilized in various civil engineering applications, such as dry masonry^1^ and columns^2^ or temporary supports consisting of stacks of cuboid elements and hydraulic jacks that are used during building rectification^3^. A distinguishing characteristic of these elements is their inability to transfer tensile stresses.

The classic nineteenth century block-stacking problem focused on the static balance of an overhanging stack of rigid elements^4^. The issue has undergone evolution over time, exemplified by the utilization of multiple elements within a single row^5^ or by incorporating elements of different lengths^6^. In^7^, it was demonstrated that a greater overhang can be achieved by considering friction forces between elements.

Ongoing research regarding dry-joint structures, focusing on their mechanical behaviour, encompasses both experimental and theoretical studies.

An analysis of multidrum columns with identical heights but varying numbers of elements revealed that the greater the number of elements is, the lower the bearing capacity of the column^8^. Tests performed on wooden^9^ and steel stacks^10^ subjected to longitudinal loading showed that their longitudinal stiffness was not constant but was dependent on the value of the longitudinal load. Tests of monotonically loaded stacks of bricks^11^, earthblocks^12^ and interlocking blocks^13^ revealed a nonlinear load–deformation relationship. Tests of multidrum columns^8^ demonstrated that the type of joint has a great influence on their load bearing capacity. In^14–16^, it was shown that an increase in longitudinal load closed the gaps visible between the elements of a dry-joint structure. Experimental determination of the contact surface area between bricks presented in^17^ showed that this area ranged from 15 to 95%, of the section of bricks, with increasing load. The authors of^18,19^ suggested the important role of geometric imperfections, which have an impact on the contact area between elements.

Tests conducted on wooden^9^ and steel stacks^10^ subjected to cyclic longitudinal loading demonstrated a hysteresis loop indicating the presence of nonconservative forces caused by internal friction. Similarly, tests involving cyclic loading^20^ of dry-joint walls made of miscanthus concrete elements resulted in noticeable hysteresis loops during the loading–unloading phase.

The analysis of temporary support structures exposed to transverse loads is particularly interesting. In the tests described in^21^, a wooden stack loaded in the transverse direction exhibited nonlinear stiffness characteristics, and cyclic loading resulted in a hysteresis loop. Laboratory tests on dry stack walls demonstrated that the sliding of elements can occur when this type of structure is subjected to an in-plane load^22^. Moreover, an investigation of a dry joint under shear force revealed that these displacements are irreversible^23^. In^24^*,* the authors showed that the appearance of a hysteresis loop during cyclic loading of dry joint walls was particularly connected with the relative displacement of the masonry units. Research on masonry structures subjected to out-of-plane loads has paid particular attention to the effect of joint opening behaviour^25^.

The computational analysis of dry stack systems often requires the use of advanced software and time-consuming calculations, such as micromodelling^26^ or dynamic simulation via the distinct element method^27–29^. This approach is viable, especially for the estimation of collapse loads and overall failure mechanisms. However, the disadvantages of these methods necessitate the development of simpler models, such as a homogenized^30^ approach. The other idea for describing mechanical behaviour is the use of a simple spring model^31^. This method is efficient but maintains the accuracy of the required results only in the case of monotonic loading; thus, it is not suitable for describing cyclic loading where a hysteresis loop occurs.

There is a research gap on the establishment of consistent simplified models for dry stack structural elements, with parameters that can be calibrated based on laboratory tests. In this study, the authors conducted research on an exemplary dry stack structural element in the form of a temporary support. The main objectives of this research were to propose a mathematical model for dry stack structural elements under cyclic bending moments and to define an algorithm for calibrating the parameters of the model. The model presented in this paper has been specifically developed for the purpose of building rectification design, which is a pioneering approach within the field of civil engineering^32^. One of the major strengths of this model lies in its universal applicability, as it can be easily adapted for use with other types of dry stack structures.

## Research programme

The main aim of the present research was to determine a mathematical model of dry stack structural elements with geometric imperfections loaded with a cyclic bending moment and to develop an algorithm for calibrating the model’s parameters based on laboratory tests. Furthermore, the influence of geometric imperfections on the transverse stiffness characteristics of the support in the *x* direction was highlighted. The transverse stiffness ${k}_{x}$ was considered according to (1), and the characteristics of the transverse stiffness were considered a function ${k}_{x}\left({Q}_{x}\right)$

1 $${k}_{x}=\frac{{\text{d}}{Q}_{x}}{{\text{d}}{u}_{x}},$$

where ${Q}_{x}$ is the transverse load imposed on the support head and ${u}_{x}$ is the transverse displacement of the support head caused by the transverse load.

The construction of the mathematical model and analysis of the transverse stiffness characteristics of the dry stack structural element required a series of tests. The results of laboratory tests conducted on a single specimen of a temporary support are shared here. This support consisted of a stack of cuboid elements and a hydraulic jack, and served as an example of a dry stack structural element.

**Figure 1:**
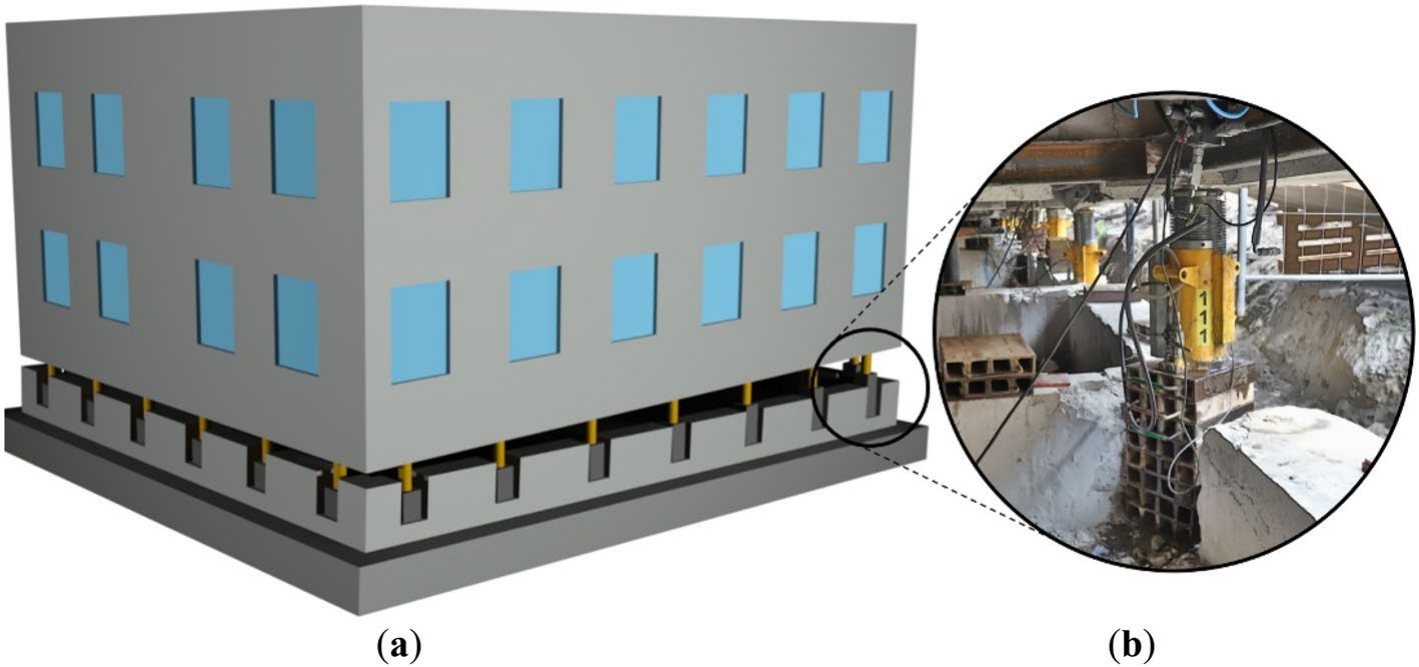
Rectification of a building: (**a**) the method of nonuniform elevation and (**b**) temporary support composed of a hydraulic jack and a stack of cuboid steel elements.

Temporary supports composed of a stack of cuboid elements and hydraulic jacks are notably applied in the rectification of vertically deflected buildings and structures. This method involves embedding hydraulic jacks into the walls of a structure to perform nonuniform lifting of the building (Fig. 1a). The elevation required in this process, which is often greater than 1 m, exceeds the typical maximum extension of the piston of a hydraulic jack, which is commonly limited to 0.2 m. To achieve the required elevation, each jack is underpinned with a proper support, commonly a stack of cuboid elements made of steel or wood. As a result, an elevated part of the rectified building rests entirely on a system of temporary supports consisting of hydraulic jacks placed on stacks of cuboid elements (Fig. 1b).

**Figure 2:**
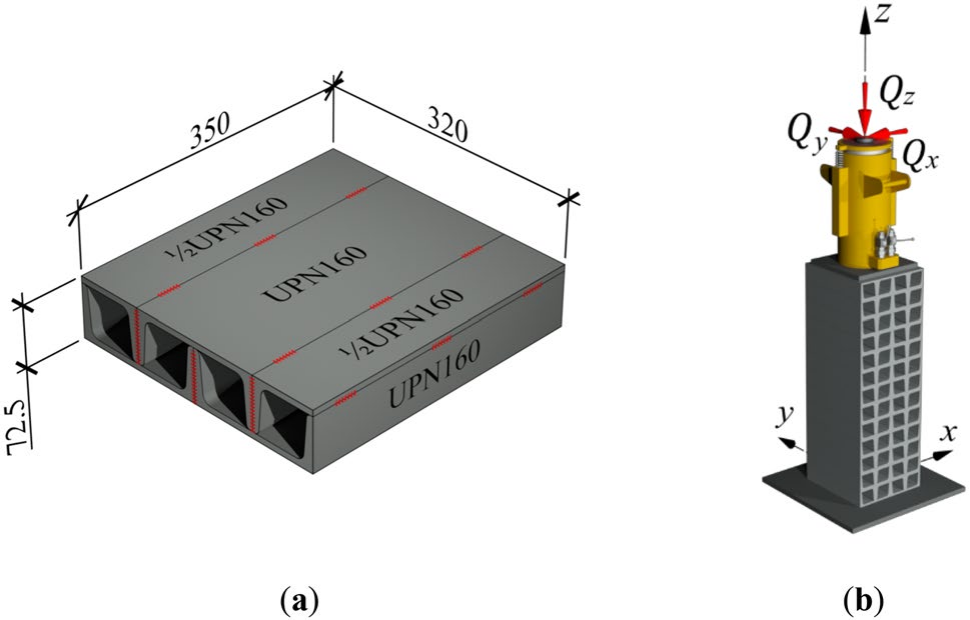
Temporary support: (**a**) a single cuboid element of the support with nonstructural welds in red; (**b**) loads imposed on the temporary support.

Each of the cuboid elements comprises three rolled profiles UPN160 and two half-profiles 1/2UPN160 (Fig. 2a) made of steel S235. The profiles forming a cuboid element are connected with one another by only nonstructural edge welds. Placed on top of each other, the cuboid elements form a stack. Under real conditions, during the rectification process, any load, including the vertical load (${Q}_{z}$) caused by the gravitational force of the building and horizontal loads (${Q}_{x}$ and ${Q}_{y}$) resulting from, for example, wind pressure and 2nd-order effects, must be transferred to the foundation only by means of the temporary support (Fig. 2b). The safety of the whole process depends mainly on the static characteristics of the temporary support, particularly the longitudinal (${k}_{z}$) and transverse (${k}_{x}$ and ${k}_{y}$) stiffness characteristics.

**Figure 3:**
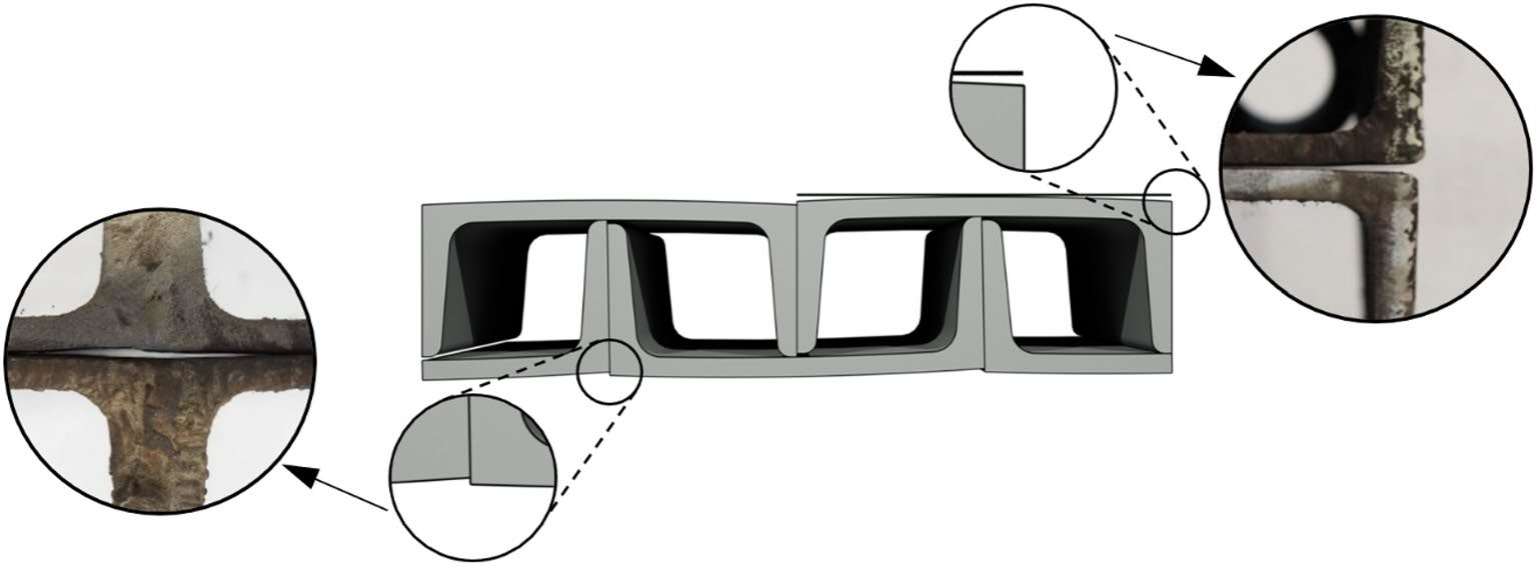
Geometric imperfections in the cuboid element: first group corresponds to the relative initial displacement of rolled profiles (left), and second group corresponds to the inaccurate contact of adjacent cuboid elements (right).

The geometric imperfections of cuboid elements have been proposed as a valid contributing factor to the nonlinearity and significant reductions in longitudinal stiffness observed in previous experimental tests^2^. These imperfections were categorized into two groups: first, those related to the relative initial displacement of rolled profiles forming a cuboid element (Fig. 3 left); second, those related to inaccurate contact of adjacent cuboid elements (Fig. 3 right). The undesirable gaps between the elements visible in Fig. 3 in the temporary support under consideration do not exceed 2 mm.

**Figure 4:**
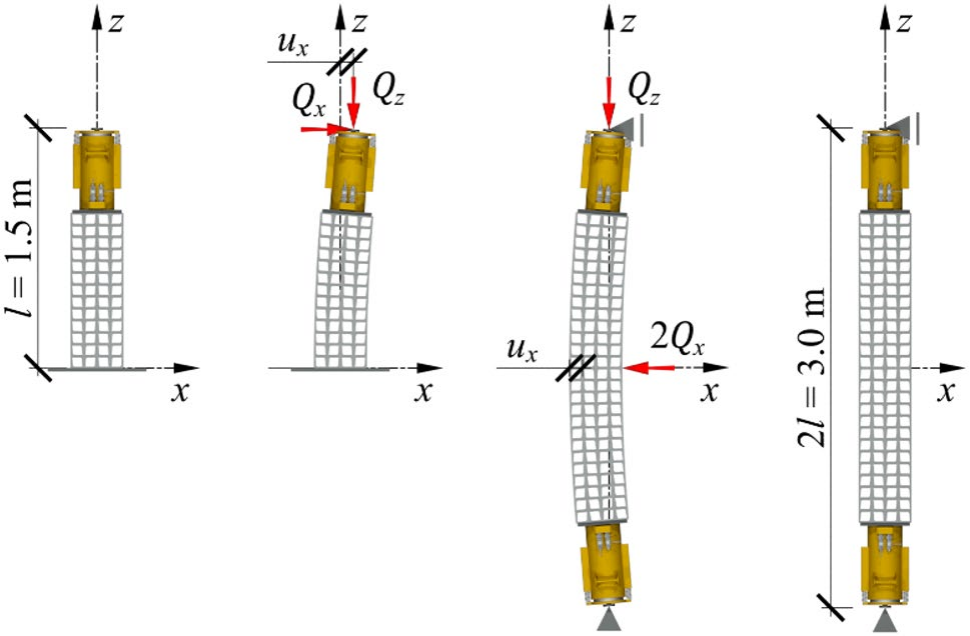
Concept of using the equivalent specimen for testing a support exposed to a transverse load. From left to right: temporary support, deformation of the loaded support, deformation of the loaded equivalent specimen, and the equivalent specimen of the support.

It was decided to test a temporary support consisting of a stack of steel cuboid elements and a hydraulic jack loaded with longitudinal and transverse forces. The tests were performed on a temporary support with a total length $l$ of 1.5 m, composed of a stack of cuboid steel elements with a length ${l}_{{\text{st}}}$ of 1 m and an axially placed hydraulic jack (Fig. 4 left). Under real conditions, the temporary support rests on a steel plate embedded in concrete grouting; hence, the support base does not move or rotate during rectification. The piston of the hydraulic jack ends with a hinge, so the bending moment is not transferred to the support. In a specific simplified case, a rod fixed at one end whose free end is loaded with two concentrated forces ${Q}_{x}$ and ${Q}_{z}$ can be considered the valid static system of the support. Force ${Q}_{z}$ is the vertical reaction balancing gravitational force, and ${Q}_{x}$ is the horizontal reaction force that balances the loads produced by the wind and 2nd-order forces resulting from the strains of adjacent supports.

For technological and safety reasons, a straightforward simulation of such a scenario under laboratory conditions was not possible. The support test would require loading the setup with a gravity force of up to 500 kN and a variable transverse force while simultaneously providing free movement of the free end of the support exposed to these forces. Instead, the experiments utilized a setup consisting of twenty-seven cuboid elements and two hydraulic jacks to create two identical supports, with their bases in contact. The response of such an equivalent specimen subjected to a midspan load of ${2Q}_{x}$ was found to mirror the actual behaviour of a support loaded with a force of ${Q}_{x}$ (Fig. 4).

To determine the elements and parameters of the model, a series of tests were planned. Initially, the support was loaded with a longitudinal force equivalent to the vertical reaction force taken by the support ${Q}_{z}$. Then, it was loaded and unloaded with a force ${Q}_{x}$ of positive and negative values. Under the aforementioned loading conditions, the analysed specimen underwent transverse displacement ${u}_{x}$ at the midspan.

**Figure 5:**
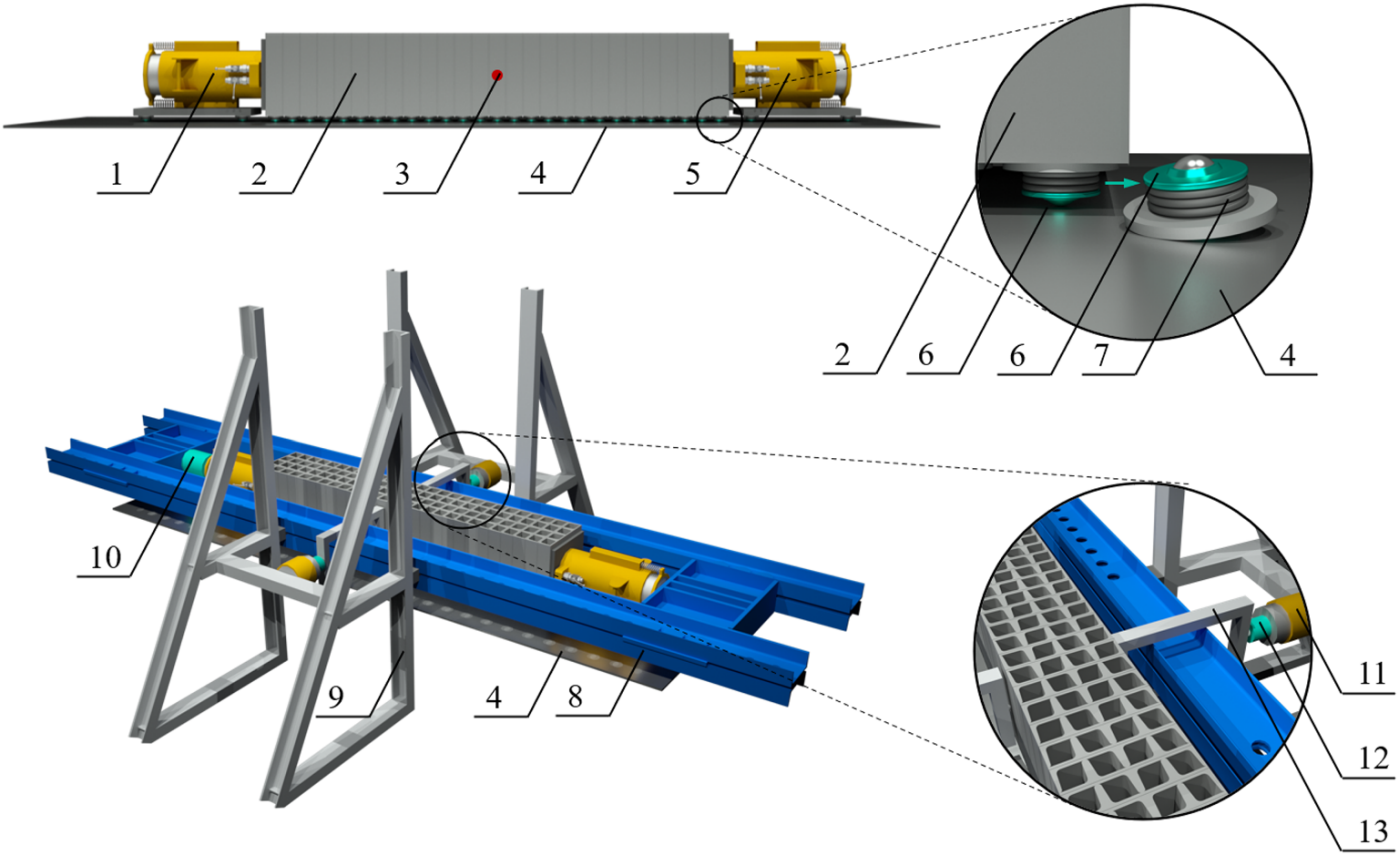
Test setup: passive hydraulic jack (1), stack of cuboid elements (2), displacement measuring point (3), steel platform (4), active hydraulic jack (5), ball transfer unit (6), rubber spring suspension (7), steel frame (8), buffer stop (9), load cell (10), horizontal actuator (11), load cell (12), and force transferring element (13).

It was decided to test the specimens (1, 2, 5—Fig. 5) in a horizontal position. The specimen rested on a platform made of polished steel (4—Fig. 5). Owing to the suspended ball transfer units (6, 7—Fig. 5), the displacements and deformations of the specimen in the *x*, *y* and *z* directions during the tests were unrestrained. Other elements of the test setup included a steel frame (8—Fig. 5) acting as the tie rod, steel buffer stops (9—Fig. 5), and elements transmitting a transverse load to the specimen (13—Fig. 5).

The gravity force ${Q}_{z}$ loading the real support was represented in the equivalent specimen by an active hydraulic jack operating along the support axis (5—Fig. 5). The pistons of both hydraulic jacks (1, 5—Fig. 5) were ended with hinges. Simultaneously, the transverse force ${Q}_{x}$ loading the real support was represented in the equivalent specimen by two horizontal actuators operating perpendicularly to the support axis (11—Fig. 5). The longitudinal load ${Q}_{z}$ and transverse load ${Q}_{x}$, as well as the transverse displacement ${u}_{x}$ at the midspan of the equivalent specimen, were continuously measured during the tests (10, 12, 3—Fig. 5).

**Figure 6:**
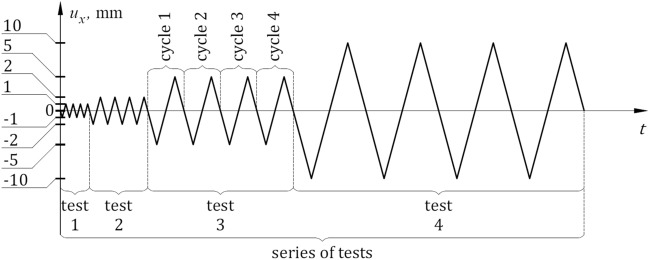
Cycle, test and series of tests.

The following terms used in this paper are defined as follows:

- Loading‒unloading cycle with force ${Q}_{x}$ with positive and negative values—a cycle consisted of loading and unloading the system within a range of horizontal displacements ${u}_{x}$ specified for a particular cycle, changing in the following way: $0 \to {u}_{x,{\text{min}}} \to 0\to {u}_{x,{\text{max}}}\to 0$ (Fig. 6).
- Test—four load cycles, one after another, characterized by set extreme values ${u}_{x,{\text{extr}}}={u}_{x,{\text{max}}}=-{u}_{x,{\text{min}}}$ (Fig. 6).
- A series of tests—four tests performed sequentially, wherein each test corresponded to a different value of ${u}_{x,{\text{extr}}}$ (Fig. 6).

A series of tests of the support was conducted with a longitudinal force ${Q}_{z}$ = 500 kN. The particular tests covered by series were characterized by displacements ${u}_{x,{\text{extr}}}$ of 1 mm, 2 mm, 5 mm, and 10 mm. The research programme is shown in Table 1.

**Table 1:** Research programme.

Series of tests name	Test name	${Q}_{z}$(kN)	${u}_{x,{\text{extr}}}$(mm)	Number of cycles
B 500	B 500/1	500	1	4
	B 500/2	500	2	4
	B 500/5	500	5	4
	B 500/10	500	10	4

## Test procedure

**Figure 7:**
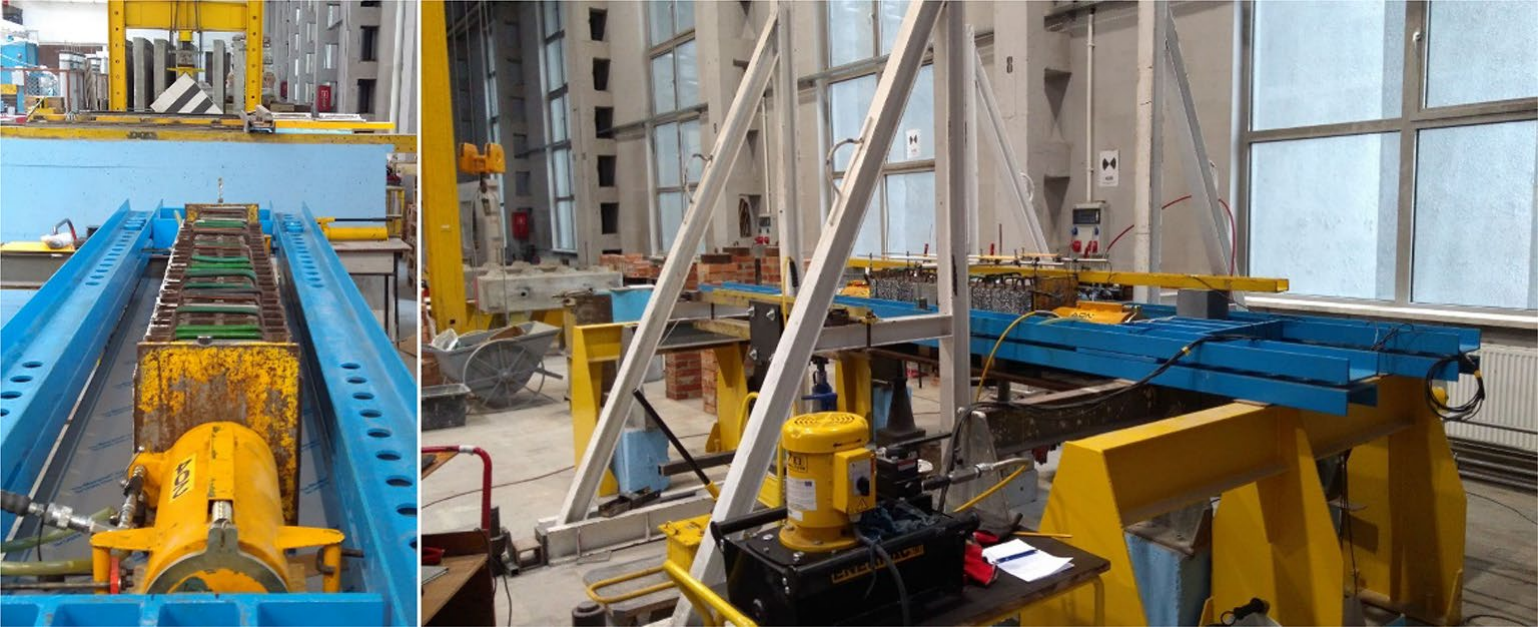
Test setup.

The tests were performed with the test setup illustrated in Fig. 7. The measuring system consisted of an analogue-to-digital converter (PPUH “Z-TECH” Zbigniew Jura) working in conjunction with a PC running proper software for data acquisition. The measurement data were acquired at a frequency of 2 Hz. Longitudinal and transverse loads were measured by means of load cells (class 0.5) providing a 1000 kN capacity for the longitudinal force ${Q}_{z}$ and a 250 kN capacity for the transverse force ${Q}_{x}$. The horizontal displacement ${u}_{x}$ at the midspan of the equivalent specimen was measured both during the loading phase of the support with longitudinal force and during the loading phase of the support with transverse force by a linear variable differential transducer (class 0.2) with a measurement range of ± 50 mm (Peltron PSz50).

**Figure 8:**
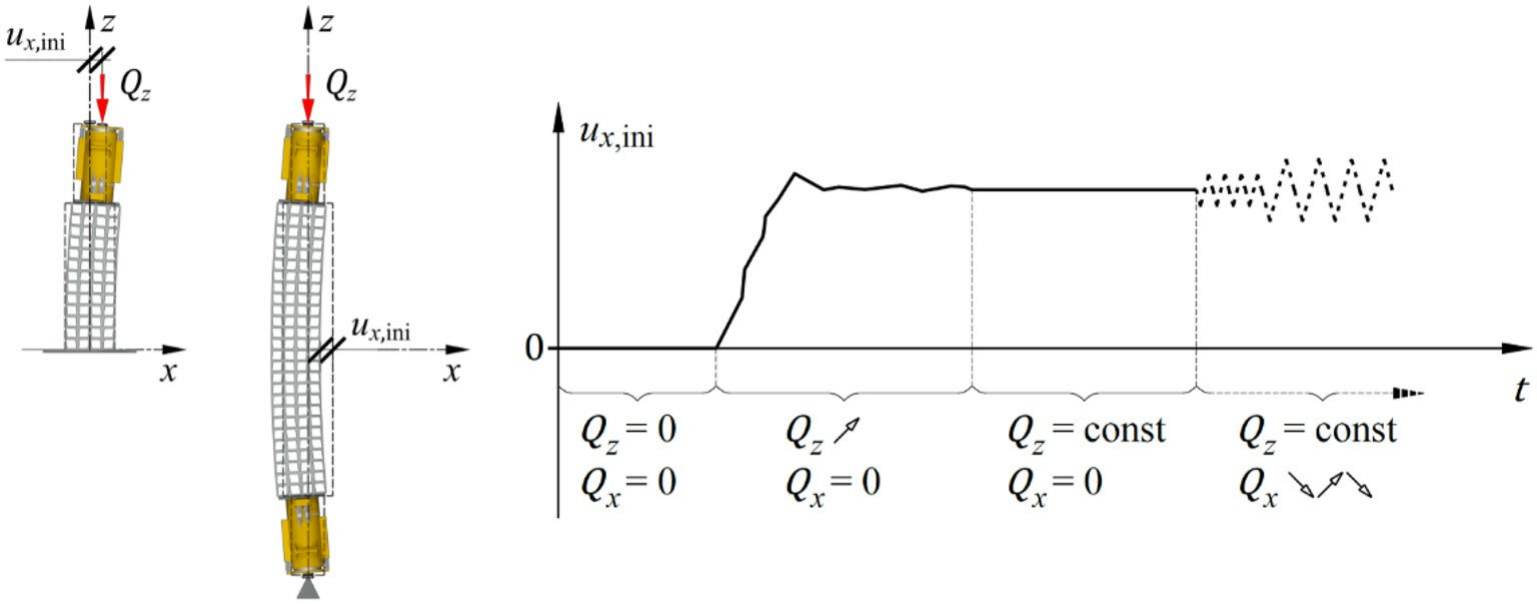
Transverse displacement caused by longitudinal load (steady state).

A series of tests was preceded by a precise, coaxial arrangement of the stack elements and jacks. Subsequently, the longitudinal load ${Q}_{z}$ was gradually applied to the specimen up to a value of 500 kN. The specimen was subjected to an increasing longitudinal load and experienced deformation. At a constant longitudinal load ${Q}_{z}$_,_ the support demonstrated a certain change in its length and a certain deflection ${u}_{x,{\text{ini}}}$. At a constant longitudinal load, the support exhibited significant variability in both its length change and deflection ${u}_{x,{\text{ini}}}$. The deflection in the *yz*-plane was negligible (less than 3 mm). This condition, under a constant longitudinal load (disregarding certain rheological phenomena in the material), remained unchanged over time and was defined as the steady state (Fig. 8).

**Figure 9:**
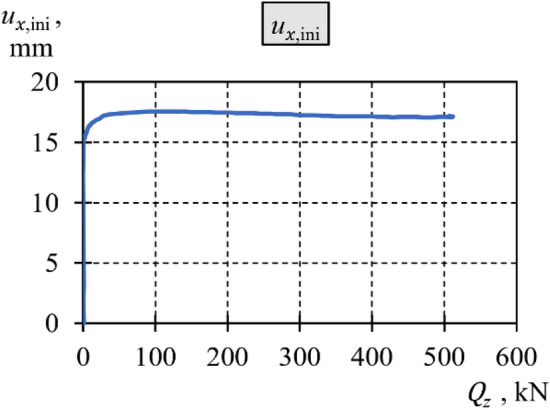
Diagram of transverse displacement ${u}_{x,{\text{ini}}}$ as a function of longitudinal force ${Q}_{z}$.

The deflection resulting from loading the support solely in the longitudinal direction was measured to be 17.1 mm (${u}_{x,{\text{ini}}}/l$ = 1/175). Under ideal conditions, this lateral deflection does not occur. Under actual operating conditions, the value of ${u}_{x,{\text{ini}}}$ is determined by a combination of geometric imperfections of the cuboid elements resulting in overall curvature of the stack’s axis, along with some additional 2nd-order effects. After more in-depth analysis of the deflection under the longitudinal force (Fig. 9), it was observed that the ${u}_{x,{\text{ini}}}$ value was established at a relatively low level of longitudinal force (${Q}_{z}\hspace{0.17em}$ = ~ 25 kN), and its changes during the further incremental increase in the longitudinal force were negligible (under 0.5 mm, see Fig. 9). Thus, it can be concluded that the main reason for the ${u}_{x,{\text{ini}}}$ value was the presence of geometric imperfections in the cuboid elements.

**Figure 10:**
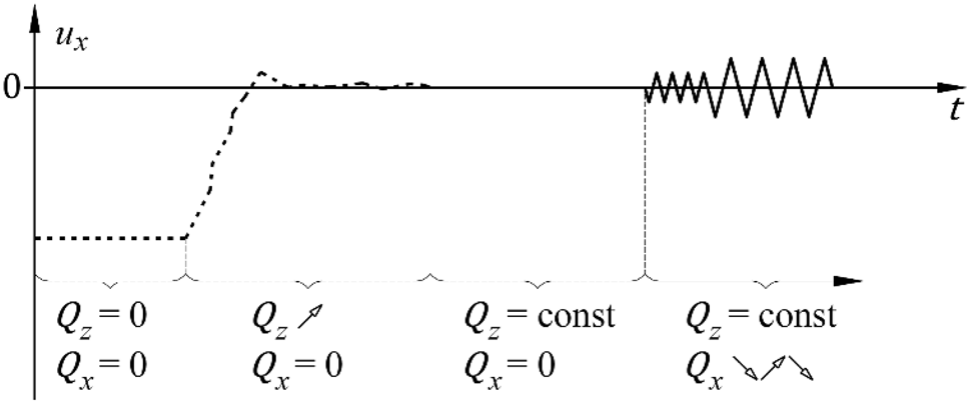
New initial position.

The previously mentioned steady state defines the support’s equilibrium position at a specific constant value of longitudinal load. This established the primary reference point for all subsequent tests in the series, represented as a new initial position (Fig. 10).

When the temporary support was subjected to a constant longitudinal force, a series of tests was conducted in which the transverse load was incrementally adjusted to induce transverse displacement at a rate of approximately 0.2 mm/s. The transverse loads, along with the transverse displacement, were continuously monitored throughout the tests.

## Results

**Figure 11:**
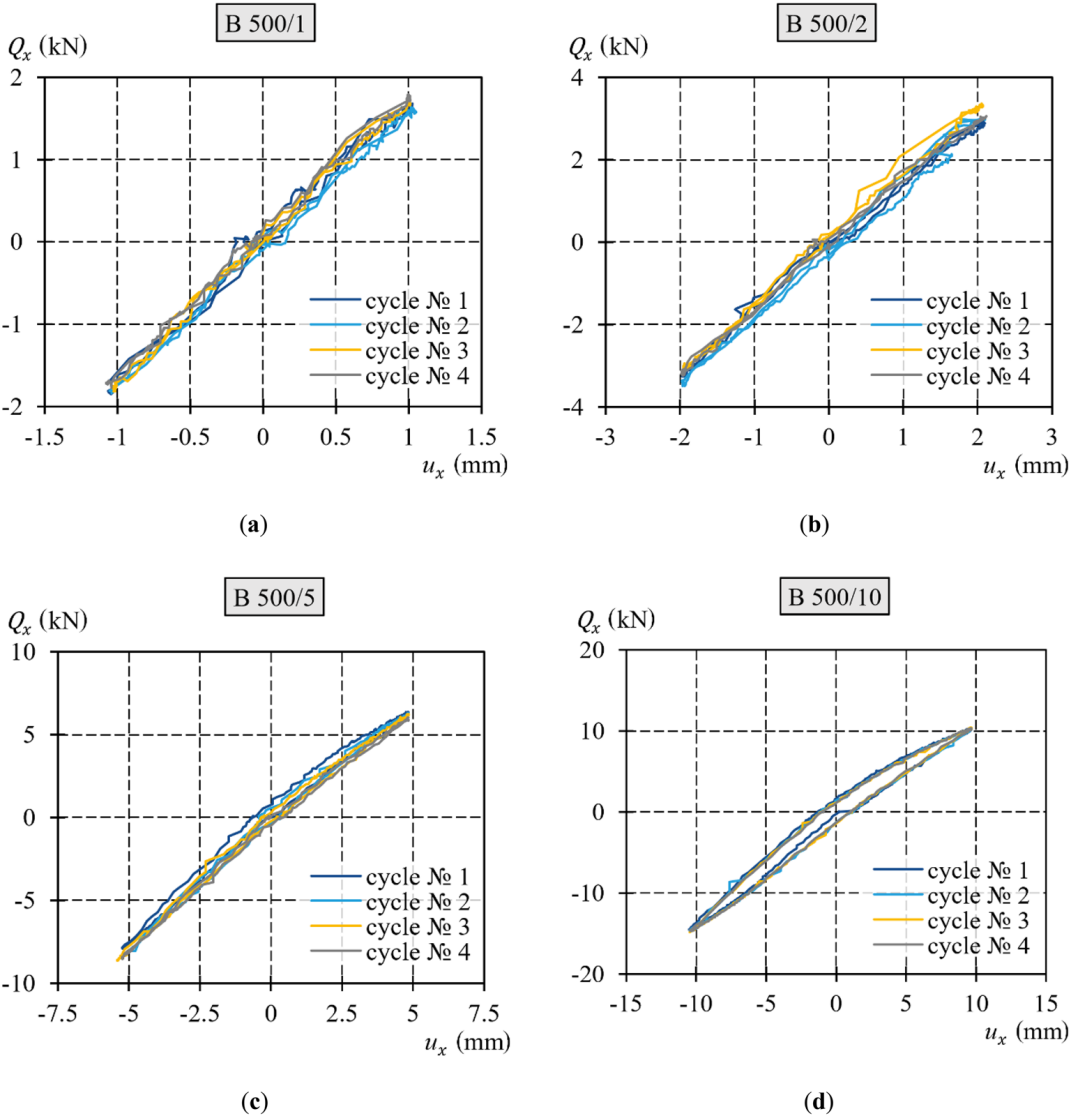
Diagrams of the transverse load–transverse displacement relationship of the extreme values: (**a**) ± 1 mm; (**b**) ± 2 mm; (**c**) ± 5 mm; (**d**) ± 10 mm.

Figure 11 presents diagrams of the transverse load against the transverse displacement obtained from testing the support. The extreme transverse displacements (${u}_{x,{\text{extr}}}$) in these tests were equal to 1, 2, 5, and 10 mm, according to Table 1. Figure 10 shows the values determined with reference to the new initial position. The following four loading‒unloading cycles with transverse forces of positive and negative values are delineated by dark blue, yellow, blue, and grey lines, respectively. In each of the tests, the diagrams for cycles 2–4 were nearly identical, as consecutive loops nearly overlapped. Furthermore, the increase in transverse displacement resulted in a more apparent hysteresis loop and nonlinear trajectory of the upper and lower branches.

**Figure 12:**
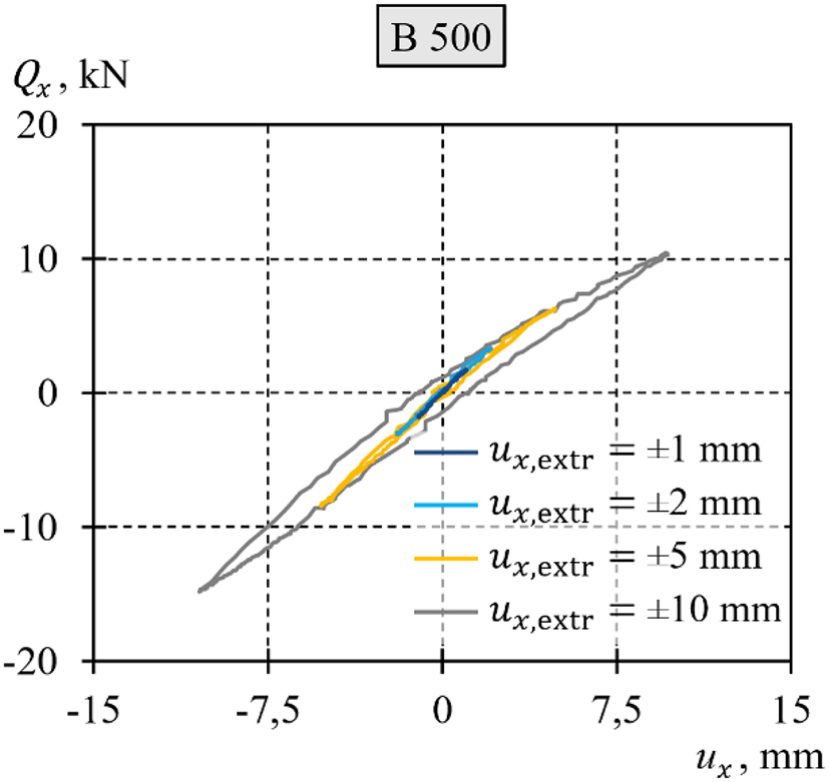
Comparison of diagrams of transverse load–transverse displacement at the end of the support.

To summarize the results illustrated in Fig. 11, particular diagrams for different tests were replicated for comparison in Fig. 12. Hysteresis loops, depicted in a singular diagram, acquired from the tests with a smaller transverse displacement, were contained within the loops resulting from the tests with a greater displacement. The permanent displacement demonstrated an increase corresponding to the increasing extremum displacement. The loops were not symmetric, and their asymmetry increased with increasing extreme displacement ${u}_{x,{\text{extr}}}$.

For each test, the diagrams of the second, third, and fourth cycles of loading‒unloading with transverse force were generally identical and overlapped. Considering the above, further considerations were based on singular diagrams of loading‒unloading cycles, which are representative of a given test.

**Figure 13:**
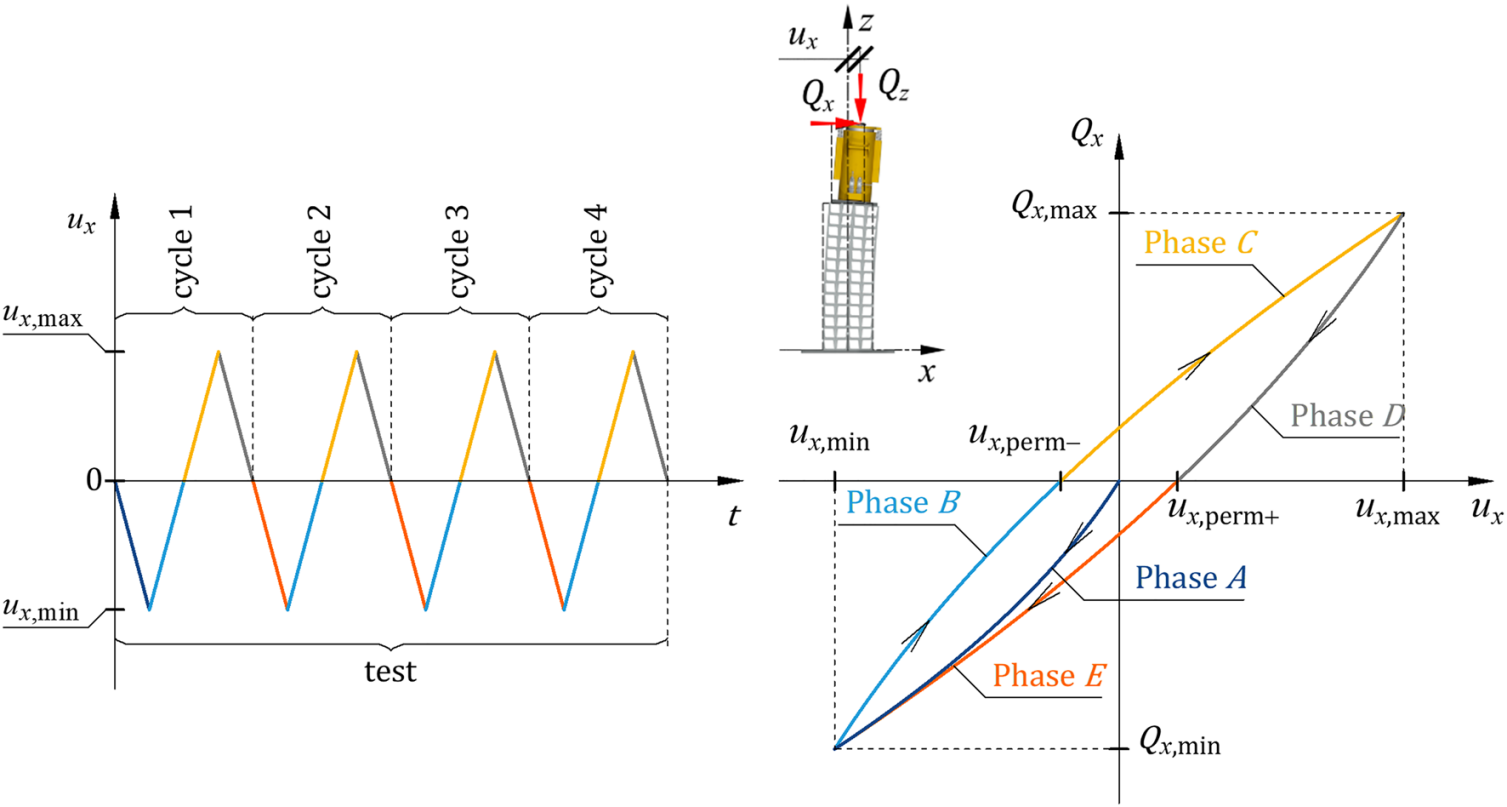
Phases identified in the loading‒unloading cycle.

The diagrams from the different tests presented in Fig. 12 have common characteristics. In each test, the permanent displacement increased as the extreme horizontal displacement increased. Greater transverse displacement resulted in a larger hysteresis loop with more apparent asymmetry. Five characteristic phases in the hysteresis loop branches were defined. These phases corresponded to the characteristic stages of the cyclic loading test (Fig. 13):

- Phase *A* corresponded to the monotonic loading of the system, wherein the force ${Q}_{x}$ was applied along the negative *x* axis. The force was gradually increased from zero to a certain ${Q}_{x,{\text{min}}}$ value, at which the midspan of the equivalent specimen attained the displacement ${u}_{x,{\text{min}}}$ required for this test. During this phase, the ${Q}_{x}-{u}_{x}$ relationship demonstrated nonlinearity, whereby an increase in the load ${Q}_{x}$ resulted in a change in the slope of the curve.
- Phase *B* corresponded to the stage of monotonic unloading, which concluded that when the force ${Q}_{x}$ ceased to act, the permanent deformation ${u}_{x,{\text{perm}}-}$ became evident. During this phase, the relationship ${Q}_{x}-{u}_{x}$ was nonlinear, as a decrease in the load ${Q}_{x}$ resulted in a change in the slope of the curve.
- Phase *C* corresponded to the monotonic loading of the system, wherein the force ${Q}_{x}$ was applied along the positive *x* axis. The force was gradually increased from zero to a certain ${Q}_{x,{\text{max}}}$ value at which the midspan of the equivalent specimen attained the displacement ${u}_{x,{\text{max}}}$ required for this test. During this phase, the ${Q}_{x}-{u}_{x}$ relationship exhibited nonlinearity: an increase in the load ${Q}_{x}$ led to a change in the slope of the curve.
- Phase *D* corresponded to the stage of monotonic unloading, which concluded that when the force ${Q}_{x}$ ceased to act, the permanent deformation ${u}_{x,{\text{perm}}+}$ became evident. During this phase, the relationship ${Q}_{x}-{u}_{x}$ was nonlinear, as a decrease in the load ${Q}_{x}$ resulted in a change in the slope of the curve.
- Phase *E* corresponded to the monotonic loading of the system wherein the force ${Q}_{x}$ was applied along the negative *x* axis The force was gradually increased from zero to a certain ${Q}_{x,{\text{min}}}$ value at which the midspan of the equivalent specimen attained the displacement ${u}_{x,{\text{min}}}$ required for this test. During this phase, the ${Q}_{x}-{u}_{x}$ relationship exhibited nonlinearity, whereby an increase in the load ${Q}_{x}$ resulted in a change in the slope of the curve. In this phase, the hysteresis loop was completed.

The analysed specimen did not return to its original state upon complete unloading after being subjected to the forces ${Q}_{x,{\text{min}}}$ (to which the displacement ${u}_{x,{\text{min}}}$ corresponds) or ${Q}_{x,{\text{max}}}$ (${u}_{x,{\text{max}}}$). Instead, a noticeable permanent displacement of ${u}_{x,{\text{perm}}-}$ or ${u}_{x,{\text{perm}}+}$ was observed. The ${Q}_{x}-{u}_{x}$ diagrams were not linear, which was especially noticeable in the tests where the displacement ${u}_{x,{\text{extr}}}$ exceeded 2 mm. This indicated that the stiffness of the analysed system was not constant. The presence of nonconservative friction forces in the system was suggested by the presence of an apparent hysteresis loop.

## Model of the dry stack structural element

In line with the objectives of the present study, the authors proposed a simple model for a dry stack structure. The parameters of this model were determined based on reported research. Therefore, in defining the model, the authors adopted the following main principles:

- The ability to provide a physical interpretation of each element of the model;
- The ability to accurately describe the hysteresis loop observed in laboratory tests of dry stack structural elements subjected to cyclic bending moments;
- The ability to use a straightforward calibration algorithm for the model’s parameters, which is based on the results of laboratory tests; and
- The potential for further development, particularly in terms of parametric analysis.

The support itself consisted of nonideal elements with geometric imperfections that had an effect on the stiffness parameters of the support. As previously mentioned, these imperfections can be categorized into two distinct groups: the first group pertains to the occurrence of relative displacements of the rolled profiles forming a cuboid element, whereas the second group is related to inaccurate contact between adjacent cuboid elements.

The support took an initial position after loading with the longitudinal force ${Q}_{z}$ (Fig. 8). This position resulted from the distribution of relative interactions between adjacent cuboid elements and between the rolled profiles inside these elements, which is specific to this load. The application of an additional transverse load ${Q}_{x}$ caused a change in this steady distribution. The relationship ${Q}_{x}-{u}_{x}$ was affected by parameters of the temporary support’s material and geometry, including imperfections.

The authors proposed to present the displacement *u*_*x*_ of the dry stack structural elements as a sum of displacement components ${u}_{{\text{teor}},x}$, ${u}_{{\text{int}},x}$, ${u}_{{\text{con}},x}$, in the form of elastic deformations (2):

2 $${u}_{x}={u}_{{\text{teor}},x}+{u}_{{\text{int}},x}+ {u}_{{\text{con}},x},$$

where.

${u}_{{\text{teor}},x}$ is the displacement resulting from the idealized element’s parameters (idealized geometry and material properties),

${u}_{{\text{int}},x}$ is the displacement resulting from the microslips (effect of relative initial displacement of rolled profiles forming a cuboid element; first group of imperfections), and. ${u}_{{\text{con}},x}$ is the displacement caused by closing gaps (the effect related to inaccurate contact of adjacent cuboid elements; second group of imperfections).

**Figure 14:**
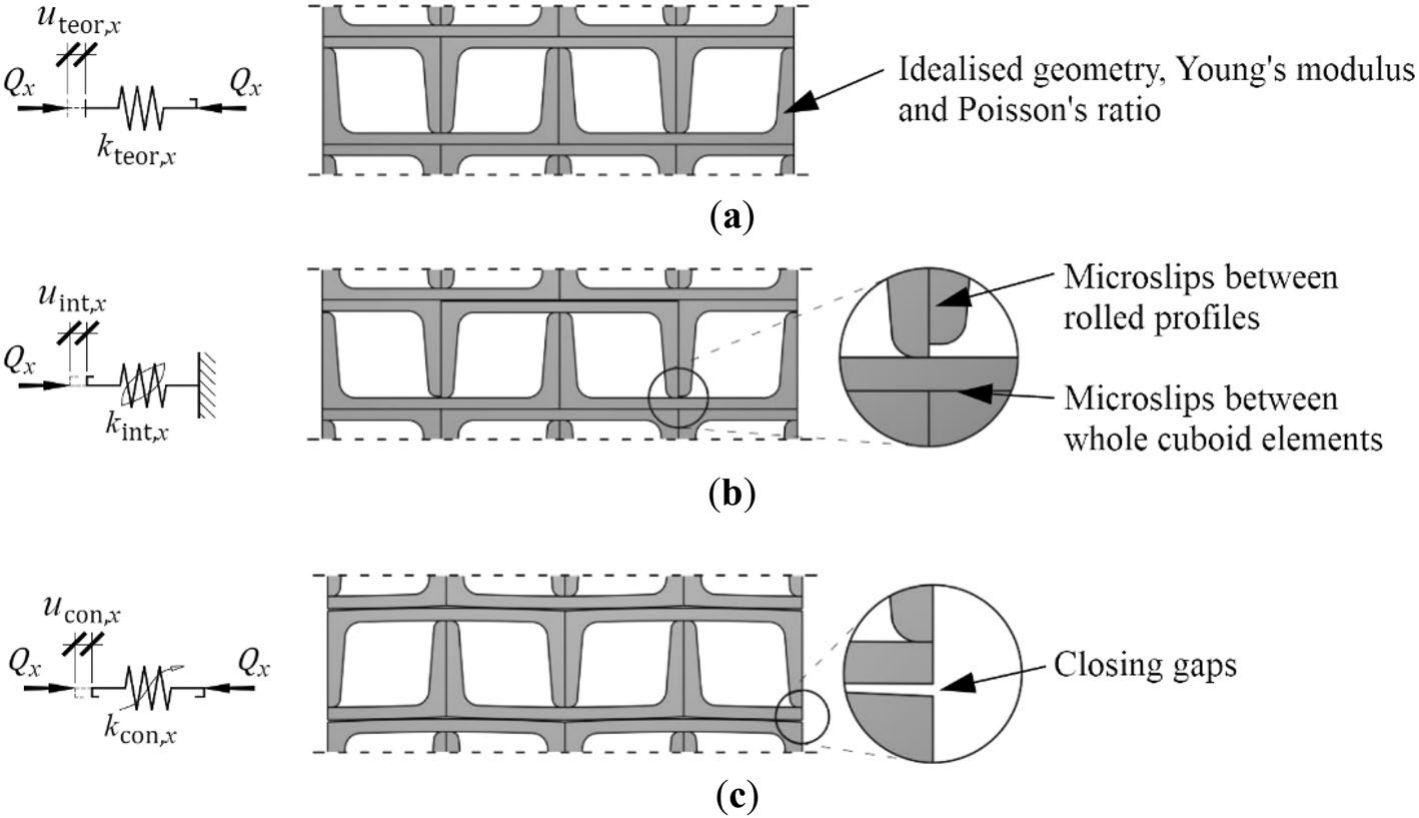
Elements of the support model exposed to transverse load: (**a**) spring ${k}_{{\text{teor}},x}$; (**b**) spring with hysteresis ${k}_{{\text{int}},x}$; (**c**) spring ${k}_{{\text{con}},x}$.

Each of the displacement components was identified in the model as the deformation of a spring. These springs were denoted as ${k}_{{\text{teor}},x}$,${k}_{{\text{int}},x}$ and ${k}_{{\text{con}},x}$, respectively. In the case of temporary support, the spring ${k}_{{\text{teor}},x}$ corresponded to the stiffness of the idealized support without any imperfections (Fig. 14a). Two additional elements included the effect of the structural solution (dry joints) and imperfections, that is, the spring with hysteresis ${k}_{{\text{int}},x}$, which represents the effect of microslip motions in the support (Fig. 14b), and the spring ${k}_{{\text{con}},x}$, which represents the effect of closing gaps (Fig. 14c).

**Figure 15:**
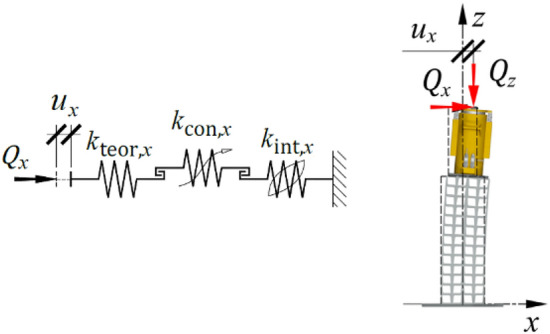
Model of the support exposed to transverse load.

Due to (2), the springs were connected in series, as illustrated in Fig. 15.

The following subsections provide detailed descriptions of each individual element of the model, along with their physical interpretation. Additionally, equations expressing the characteristics of each element are presented.

### Displacement resulting from the idealized element’s parameters

**Figure 16:**
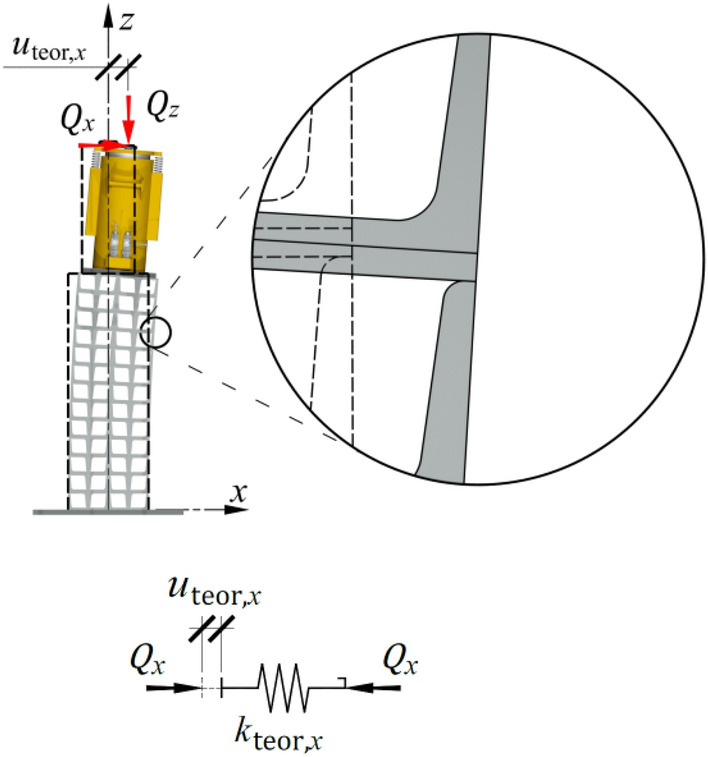
Model of the idealized support.

The horizontal displacement of the top of the idealized support, denoted as ${u}_{{\text{teor}},x}$, was caused by the elastic deformation of the material under load ${Q}_{x}$ (Fig. 16). That displacement showed a linear dependence on the load ${Q}_{x}$, and in the model, it corresponded to the elastic deformation of the spring with stiffness ${k}_{{\text{teor}},x}$, which in turn was associated with the bending of an idealized bar. Thus, the transverse stiffness ${k}_{{\text{teor}},x}$ was constant and did not include the structural solution for the support, which consisted of separate cuboid elements and did not account for geometric imperfections of the cuboid elements.

To determine the theoretical stiffness of the support, an analytical model (for example, a cantilever) or a finite element method model of an idealized structure should be used to obtain the stiffness, which is defined as the relation between force and displacement, (3). The structural elements in this model should have nominal dimensions, material constants defined in standards, and a geometry without imperfections.

In the analysed temporary support,

3 $${k}_{{\text{teor}},x}=\frac{{Q}_{x}}{{u}_{x}}.$$

The displacement ${u}_{{\text{teor}},x}$ of the model of the idealized support regarded as a single element with stiffness ${k}_{{\text{teor}},x}$ loaded with the force of value ${Q}_{x}$ was equal to:

4 $${u}_{{\text{teor}},x}=\frac{{Q}_{x}}{{k}_{{\text{teor}},x}} .$$

Relationship (4) was applicable for both positive and negative values of ${Q}_{x}$.

### Deformations resulting from microslips

**Figure 17:**
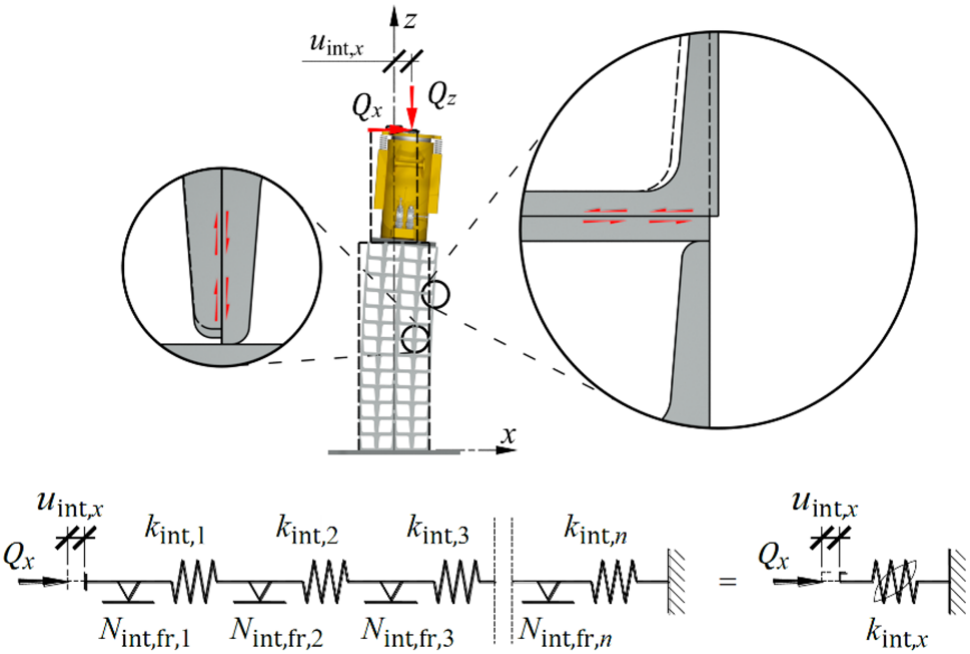
A part of support model representing the first group of imperfections.

Applying an additional horizontal force ${Q}_{x}$ to an already compressed support resulted in a bending moment and shear force. An increasing bending moment caused relative displacements of the profiles, forming cuboidal elements (Fig. 17, top left). These microslip motions resulted from the first group of imperfections—the relative initial displacement of the rolled profiles forming the single cuboid element (Fig. 3, left). An increasing shear force resulted in relative displacement of whole cuboid elements (Fig. 17, top right). These microslip motions resulted from the structural solution (dry joints without interlocking elements). The aforementioned microslip motion formed a additional partial horizontal displacement ${u}_{{\text{int}},x}$. The internal friction forces that play a role during these displacements are denoted as ${N}_{{\text{int}},{\text{fr}}}$. The above mechanisms caused a stiffness reduction in the support in the transverse direction and determined the formation of the hysteresis loop. The impact of microslips was included in the model by spring with hysteresis (Fig. 15). There was a physical interpretation for this element. It was associated with a series of alternating springs ${k}_{{\text{int}},i,x}$ and elements with friction ${N}_{{\text{int}},{\text{fr}},i}$ (index *i* refers to the number of subsequent element in the series; Fig. 17, bottom). A series of elements ${k}_{{\text{int}},i}$ and ${N}_{{\text{int}},{\text{fr}},i}$ represented microslips inside the cuboid elements (Fig. 17, top left) and microslips on contact surfaces of whole cuboid elements (Fig. 17, top right).

The condition required for the internal force to occur in a specific spring of the series was to overcome the friction forces in the preceding element, which acts in the direction opposite to the velocity direction induced by a change in load ${Q}_{x}$. It was assumed that in the analysed series, each *i*th spring and each *i*th element with friction were the same

5 $${k}_{{\text{int}},1}={k}_{{\text{int}},2}=\cdots ={k}_{{\text{int}},i}=\cdots ={k}_{{\text{int}}},$$

6 $${N}_{{\text{int}},{\text{fr}},1}={N}_{{\text{int}},{\text{fr}},2}=\cdots ={N}_{{\text{int}},{\text{fr}},i}=\cdots ={N}_{{\text{int}},{\text{fr}}}.$$

**Figure 18:**
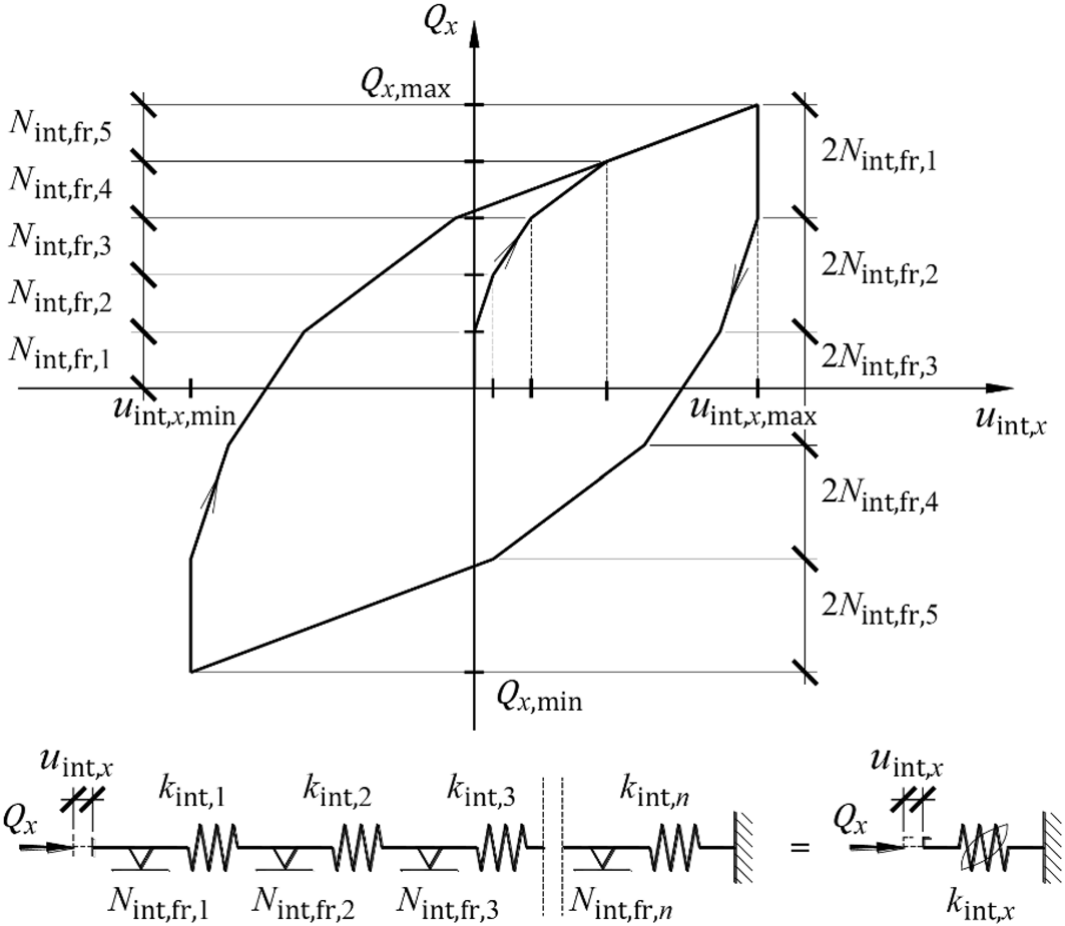
Hysteresis loop of a series of five spring elements and five elements with friction.

As there were nonconservative forces, this series during loading and unloading demonstrated a hysteresis loop. Moreover, during loading and unloading of this series, its stiffness changed in a stepwise pattern. Changes in the stiffness during the first load (phase *A*) occurred at the load values ${Q}_{x}$, which were integral multiples of the value of the friction forces ${N}_{{\text{int}},{\text{fr}}}$. During unloading and reloading (phases *B* and *C* or phases *D* and *E*), these stepwise changes were observed at even-numbered multiples of values of friction forces. The representative hysteresis loop, assuming that five spring elements work, is illustrated in Fig. 18.

For simplification, further considerations focused on positive values of both load and displacement.

#### Phase *A*

The additional index *A* in the symbols represents the quantity of phase A.

The number $p$ of spring elements under a given load ${Q}_{x}$ in phase *A* was determined from Eq. (7), in which $\lfloor \rfloor$ is the floor function

7 $$p = \lfloor \frac{{\left| {Q_{x} } \right|}}{{N_{{{\text{int}},{\text{fr}}}} }}\rfloor.$$

At a known number of loaded elements $p$, the expression for displacement in phase *A* marked as ${u}_{{\text{int}},x,A}$ can be determined for this type of system on the basis of $p$, the stiffness of a particular spring element ${k}_{{\text{int}}}$ and the friction force in a particular element with friction ${N}_{{\text{int}},{\text{fr}}}$ obtained via the sum of an arithmetic series in accordance with Eq. (8)

8 $${u}_{{\text{int}},x,A}=\frac{1}{{k}_{{\text{int}}}}\left[{Q}_{x}p-{N}_{{\text{int}},{\text{fr}}}\left({\frac{1}{2}p}^{2}+\frac{1}{2}p\right)\right].$$

A smooth function of displacement was obtained for an infinite number of loaded elements and for ${N}_{{\text{int}},{\text{fr}}}$ approaching zero. In that case, $p$ can be expressed as $p^{\prime}$

9 $${p}^{\mathrm{^{\prime}}}=\frac{{Q}_{x}}{{N}_{{\text{int}},{\text{fr}}}}.$$

By substituting the developed form $p^{\prime}$, instead of $p$, into (9), we obtain

10 $${u}_{{\text{int}},x,A}=\frac{1}{{k}_{{\text{int}}}}\left[\frac{{{Q}_{x}}^{2}}{{N}_{{\text{int}},{\text{fr}}}}-{N}_{{\text{int}},{\text{fr}}}\left(\frac{{{Q}_{x}}^{2}}{{{2N}_{{\text{int}},{\text{fr}}}}^{2}}+\frac{{Q}_{x}}{{2N}_{{\text{int}},{\text{fr}}}}\right)\right],$$

and the simplification results in

11 $${u}_{{\text{int}},x,A}=\frac{{{Q}_{x}}^{2}}{{2k}_{{\text{int}}}{N}_{{\text{int}},{\text{fr}}}}-\frac{{Q}_{x}}{{2k}_{{\text{int}}}}.$$

At this stage, it was necessary to introduce parameter ${\alpha }_{{\text{int}}}$, which is a product of spring stiffness ${k}_{{\text{int}}}$ and friction ${N}_{{\text{int}},{\text{fr}}}$

12 $${\alpha }_{{\text{int}}}={k}_{{\text{int}}}{N}_{{\text{int}},{\text{fr}}}.$$

By substituting (12) into (11), the following was obtained

13 $${u}_{{\text{int}},x,A}=\frac{{{Q}_{x}}^{2}}{{2\alpha }_{{\text{int}}}}-\frac{{Q}_{x}{N}_{{\text{int}},{\text{fr}}}}{{\alpha }_{{\text{int}}}}.$$

Considering that ${N}_{{\text{int}},{\text{fr}}}$ tends to zero,

14 $${u}_{{\text{int}},x,A}=\frac{{{Q}_{x}}^{2}}{{2\alpha }_{{\text{int}}}} .$$

The displacement ${u}_{{\text{int}},x,A}$ is a quadratic function of the loading ${Q}_{x}$ and the value of parameter ${\alpha }_{{\text{int}}}$.

After transformation, the inverse function was obtained and expressed as

15 $${Q}_{x}=\sqrt{{2\alpha }_{{\text{int}}}{u}_{{\text{int}},x,A}}.$$

The stiffness in phase *A* during the first loading is defined as ${k}_{{\text{int}},x,A}$ and determined from (16):

16 $${k}_{{\text{int}},x,A}=\frac{{\text{d}}{Q}_{x}}{{\text{d}}{u}_{{\text{int}},x,A}},$$

which is equal to

17 $${k}_{{\text{int}},x,A}=\frac{\sqrt{{2\alpha }_{{\text{int}}}}}{2\sqrt{{u}_{{\text{int}},x,A}}}.$$

After substituting (14) into (17) and taking into account the sign of the transverse load ${Q}_{x}$, the stiffness as a function of loading was obtained

18 $${k}_{{\text{int}},x,A}=\frac{{\alpha }_{{\text{int}}}}{{Q}_{x}}.$$

Thus, the stiffness is a rational function of the horizontal load ${Q}_{x}$ and the value of parameter ${\alpha }_{{\text{int}}}$.

The above relationships hold true only for loads and displacements with positive values. The equations modified to maintain validity for loads and displacements with negative values are presented below:

19 $${u}_{{\text{int}},x,A}={\text{sgn}}\left({Q}_{x}\right)\frac{{{Q}_{x}}^{2}}{{2\alpha }_{{\text{int}}}},$$

20 $${Q}_{x}={\text{sgn}}\left({u}_{{\text{int}},x,A}\right)\sqrt{{2\alpha }_{{\text{int}}}\left|{u}_{{\text{int}},x,A}\right|},$$

21 $${k}_{{\text{int}},x,A}=\frac{{\alpha }_{{\text{int}}}}{\left|{Q}_{x}\right|}.$$

#### Phases *B*, *C*, *D* and *E*

The additional index *B ÷ E* represents the quantities of phases *B, C, D* and* E*.

**Figure 19:**
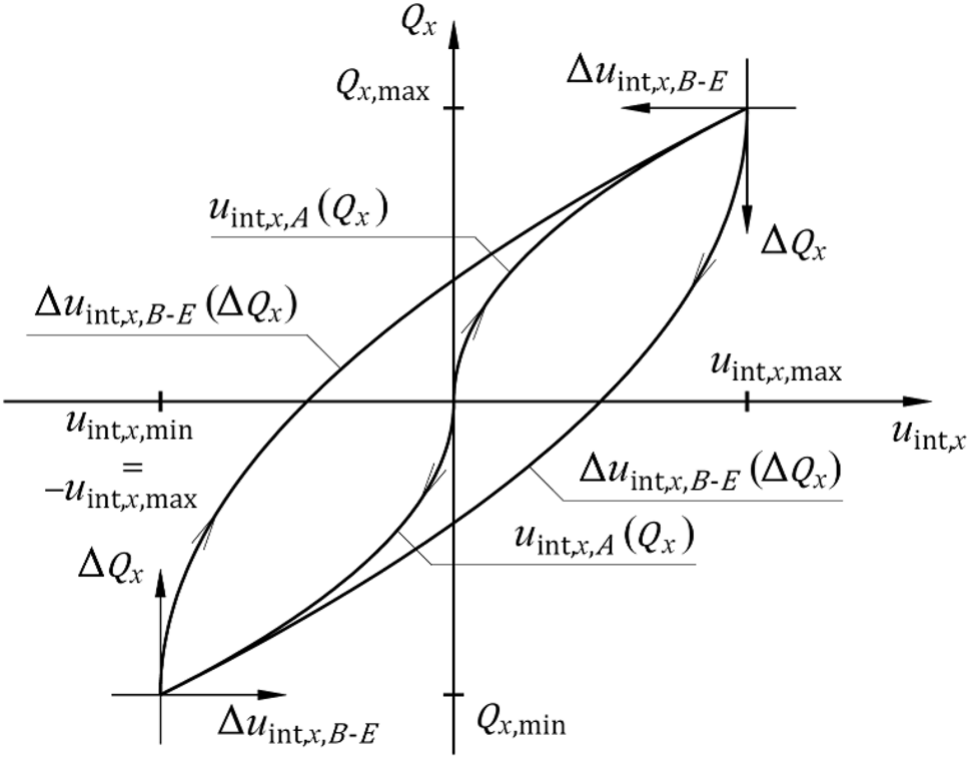
Phases *B*, *C*, *D* and *E* as functions of unloading force.

The phases of unloading and reloading (phases *B* and *C* or phases *D* and *E*) may be regarded as acting on the system with force $\Delta {Q}_{x}$ whose direction is opposite to the direction of the force exerted in the previous phase (Fig. 19). For the applied load $\left|\Delta {Q}_{x}\right|\le \left|2{Q}_{x,{\text{max}}}\right|$_,_ the series of spring elements indicates the stiffness ${k}_{{\text{int}},x,B\div E}$_,_ which is a rational function of unloading $\Delta {Q}_{x}$.

As branches of the described hysteresis loop were relative transformations in the form of reference scales, symmetry and translation, a change in horizontal displacement ${\Delta u}_{{\text{int}},x,B\div E}$ occurring in those phases was expressed by the following equation:

22 $${\Delta u}_{{\text{int}},x,B\div E}=\frac{{{\Delta Q}_{x}}^{2}}{4{\alpha }_{{\text{int}}}} ,$$

and after transformation, the reverse function is expressed as

23 $${\Delta u}_{{\text{int}},x,B\div E}=\frac{{{\Delta Q}_{x}}^{2}}{4{\alpha }_{{\text{int}}}} .$$

The stiffness during the first unloading and second loading ${k}_{{\text{int}},x,B\div E}$ is

24 $${k}_{{\text{int}},x,B\div E}=\frac{{\text{d}}{Q}_{x}}{{\text{d}}\Delta {u}_{{\text{int}},x,B\div E}},$$

which is equal to

25 $${k}_{{\text{int}},x,B\div E}=\frac{{\alpha }_{{\text{int}}}}{\sqrt{{{\alpha }_{{\text{int}}}\Delta u}_{{\text{int}},x,B\div E}}} .$$

After substituting (14) into (17), the stiffness as a function of the loading ${Q}_{x}$ was obtained:

26 $${k}_{{\text{int}},x,B\div E}=\frac{{2\alpha }_{{\text{int}}}}{{\Delta Q}_{x}}.$$

The stiffness ${k}_{{\text{int}},x,B\div E}$ in phases *B* and *C* or phases *D* and *E* is two times greater than the stiffness ${k}_{{\text{int}},x,A}$ in phase *A*.

The stiffness of the spring with hysteresis in each of the phases was expressed as rational functions (21) and (26), in which the coefficient ${\alpha }_{{\text{int}},x}$ is unknown. For a symmetric loop of hysteresis, its branches undergo relative transformations of scale, symmetry, and translation (Fig. 19). The real loops obtained from the tests were asymmetric (Fig. 11, Fig. 12). Because of the asymmetric relationship ${Q}_{x}-{u}_{x}$, the additional symbols “+” and “−” are used as subscripts in this paper. The values with the subscript “+” are related to the characteristics at positive displacements, whereas the values with the subscript “−” are related to the characteristics at negative displacements. Hence, the permanent displacements ${u}_{{\text{int}},x,{\text{perm}}+}$ and ${u}_{{\text{int}},x,{\text{perm}}-}$ (Fig. 19) for a particular hysteresis loop were:

27 $${u}_{{\text{int}},x,{\text{perm}}+}=\frac{{{Q}_{x,{\text{max}}}}^{2}}{{2\alpha }_{{\text{int}}+}}-\frac{0.25{{Q}_{x,{\text{max}}}}^{2}}{{\alpha }_{{\text{int}}+}},$$

28 $${u}_{{\text{int}},x,{\text{perm}}-}=-\left(\frac{{{Q}_{x,{\text{min}}}}^{2}}{{2\alpha }_{{\text{int}}-}}-\frac{0.25{{Q}_{x,{\text{min}}}}^{2}}{{\alpha }_{{\text{int}}-}}\right).$$

After transformation, the following equation for ${\alpha }_{{\text{int}},x}$ was obtained:

29 $${\alpha }_{{\text{int}}+}=\frac{0.25{{Q}_{x,{\text{max}}}}^{2}}{{u}_{{\text{int}},x,{\text{perm}}+}},$$

30 $${\alpha }_{{\text{int}}-}=-\left(\frac{0.25{{Q}_{x,{\text{min}}}}^{2}}{{u}_{{\text{int}},x,{\text{perm}}-}}\right).$$

### Deformations caused by closing gaps between adjacent cuboid elements

**Figure 20:**
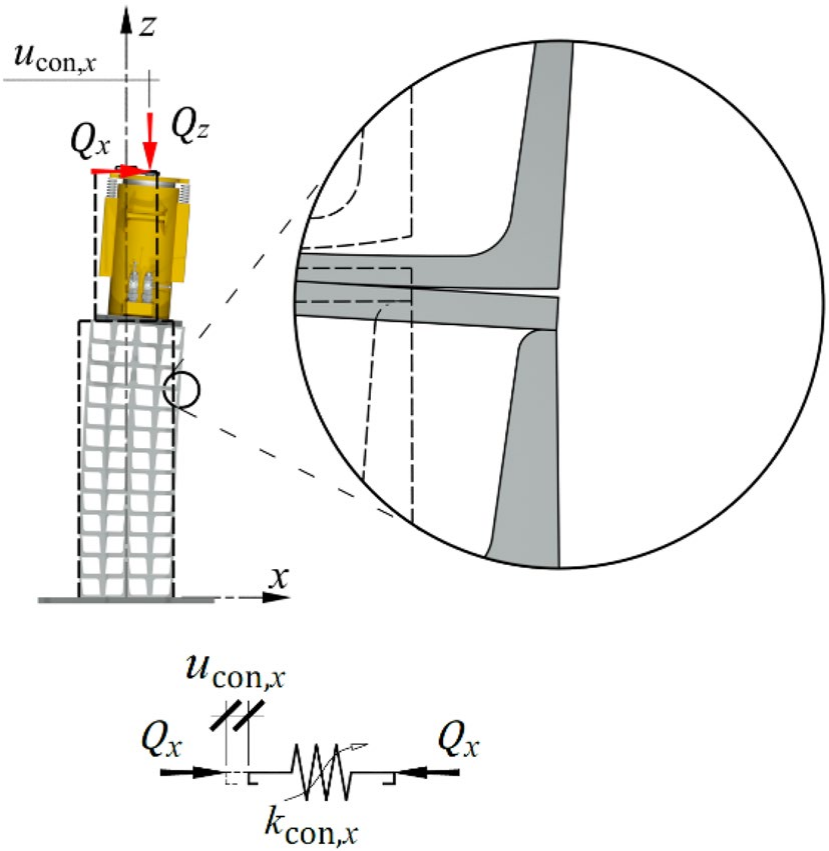
A part of support model representing the second group of imperfections.

Applying the additional horizontal force ${Q}_{x}$ to an already compressed support resulted in closing gaps and changing the areas of relative contact between the cuboid elements. These changes resulted from the second group of imperfections—inaccurate contact (Fig. 3 right). The occurrence of these imperfections in the elements decreased the stiffness of the support in the horizontal direction, and the horizontal displacement increased by ${u}_{{\text{con}},x}$ (Fig. 20). These imperfections were included in the model by adopting a nonlinear spring with stiffness ${k}_{{\text{con}},x}$.

On the basis of the observed relationship ${Q}_{x}-{u}_{x}$ obtained from the tests, the value of the elastic displacement was arbitrarily assumed to be described by an exponential relationship with two parameters ${\alpha }_{{\text{con}}}$ and ${\beta }_{{\text{con}}}$:

31 $${u}_{{\text{con}},x}={\alpha }_{{\text{con}}}\left({e}^{\frac{{Q}_{x}}{{\beta }_{{\text{con}}}}}-1\right).$$

Transformations provided the inverse function

32 $${Q}_{x}={\beta }_{{\text{con}}}{\text{ln}}\left(\frac{{u}_{{\text{con}},x}}{{\alpha }_{{\text{con}}}}+1\right).$$

The force ${Q}_{x}$ is a logarithmic function of the elastic displacement and the values of parameters ${\alpha }_{{\text{con}}}$ and ${\beta }_{{\text{con}}}$.

The function of stiffness ${k}_{{\text{con}},x}$ is determined as

33 $${k}_{{\text{con}},x}=\frac{{\text{d}}{Q}_{x}}{{\text{d}}{u}_{{\text{con}},x}}$$

was described by the following equation:

34 $${k}_{{\text{con}},x}=\frac{{\beta }_{{\text{con}}}}{{u}_{{\text{con}},x}+{\alpha }_{{\text{con}}}}.$$

By substituting (31) with (34), the stiffness as a function of the loading ${Q}_{x}$ was obtained

35 $${k}_{{\text{con}},x}=\frac{{\beta }_{{\text{con}}}}{{\alpha }_{{\text{con}}}{e}^{\frac{{Q}_{x}}{{\beta }_{{\text{con}}}}}}.$$

Thus, the stiffness ${k}_{{\text{int}},x}$ was a nonlinear function of the loading ${Q}_{x}$ and the values of parameters ${\alpha }_{{\text{con}}}$ and ${\beta }_{{\text{con}}}$.

The initial stiffness was ${k}_{{\text{con}},x,{\text{ini}}}$, and thus, the stiffness at ${Q}_{x}=$ 0 was equal to

36 $${k}_{{\text{con}},x,{\text{ini}}}={k}_{{\text{con}},x}\left({Q}_{x}=0\right)=\frac{{\beta }_{{\text{con}}}}{{\alpha }_{{\text{con}}}} .$$

The initial stiffness ${k}_{{\text{con}},x,{\text{ini}}}$ is the quotient of parameters ${\alpha }_{{\text{con}}}$ and ${\beta }_{{\text{con}}}$.

## Calibration of model parameters

In the following subsections, the parameters of the model were determined according to the test results of the temporary support, and the stiffness characteristics of the springs were compared.

### Stiffness ${k}_{\mathbf{t}\mathbf{e}\mathbf{o}\mathbf{r},{x}}$

**Figure 21:**
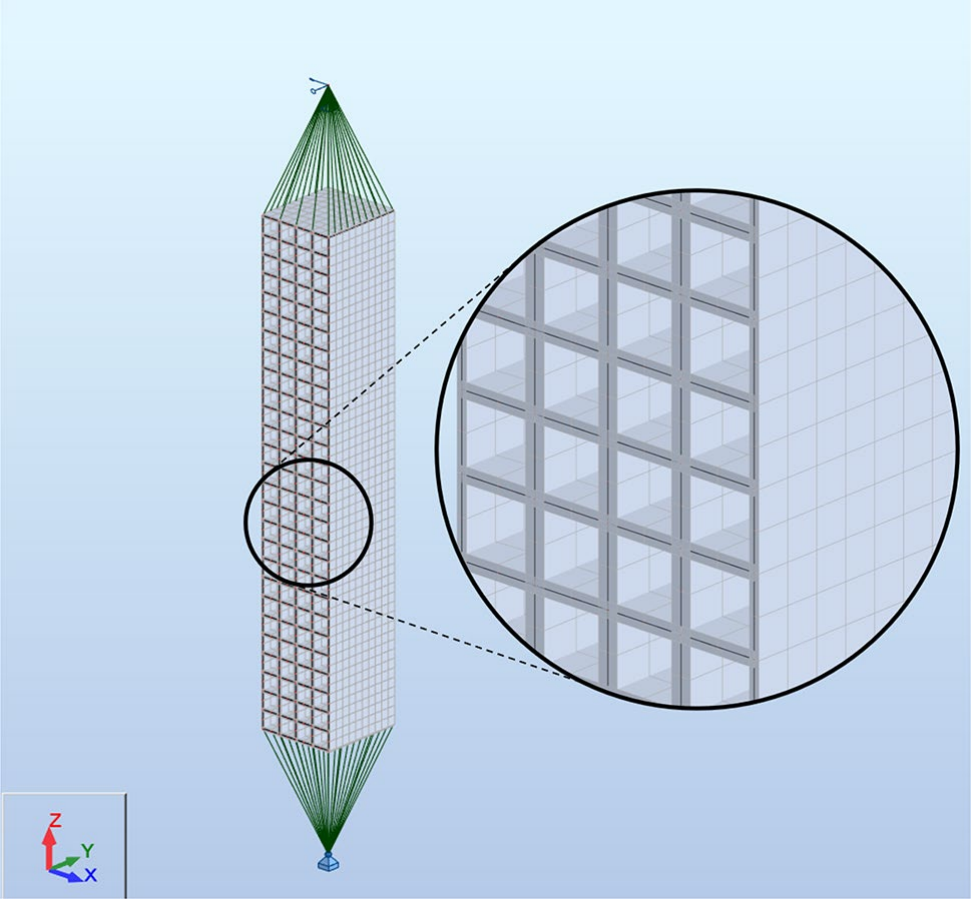
Shell model of the analysed system of the support.

**Figure 22:**
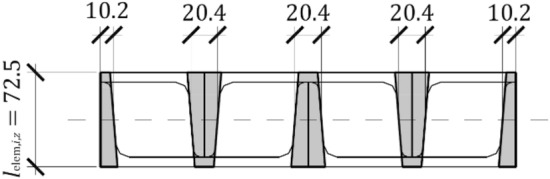
Average thickness of the shells.

To determine the stiffness of the idealized support, a shell model of the analysed system was developed using Autodesk Robot Structural Analysis Professional software (Fig. 21). Webs of adjacent cuboid elements in contact were represented by shells with double the thickness of the UPN 160 profile webs. The shells representing the flanges of the UPN profiles were modelled with an average flange thickness of UPN 160, as illustrated in Fig. 22. The elastic modulus of the material corresponding to steel S235 was 205 GPa, and a Poisson’s ratio of 0.3 was assumed. Hydraulic jacks were modelled with kinematic constraints, which are ideally stiff elements.

The model was composed of two supports: a pinned support (with rotation released around the *y*-axis) and a roller support (with rotation released around the *y*-axis and displacement along the *z*-axis), which represented the support conditions of the analysed equivalent specimen in which the jack heads ended with hinges. The load was represented by a concentrated load $Q_{x}$ = 100 kN; however, it was uniformly distributed by the width of the support through kinematic constraints. Four-node quadrilaterals finite elements were used.

The deflection ${u}_{{\text{teor}},x}$ of the load ${Q}_{x}$ applied to the model was used to determine the theoretical transverse stiffness of the analysed system in accordance with (3). The values of ${k}_{{\text{teor}},x-}$ and ${k}_{{\text{teor}},x+}$ were constant and equal to 37.84 kN/mm.

### Stiffness ${{\varvec{k}}}_{\mathbf{i}\mathbf{n}\mathbf{t},{\varvec{x}}}$ and parameter ${\boldsymbol{\alpha }}_{\mathbf{i}\mathbf{n}\mathbf{t}}$

Considering that ${u}_{{\text{int}},x,{\text{perm}}+}$ = ${u}_{x,{\text{perm}}+}$ and ${u}_{{\text{int}},x,{\text{perm}}-}$ = ${u}_{x,{\text{perm}}-}$ (Fig. 13 and Fig. 19), coefficients ${\alpha }_{{\text{int}}+}$ and ${\alpha }_{{\text{int}}-}$ were calculated from the permanent displacement obtained from the tests, which was observed when the exerted extreme force ${Q}_{x,{\text{extr}}}$ stopped. The coefficients determined as specified in (29) and (30) were ${\alpha }_{{\text{int}}-}$ = 56.68 and ${\alpha }_{{\text{int}}+}$ = 30.61.

**Figure 23:**
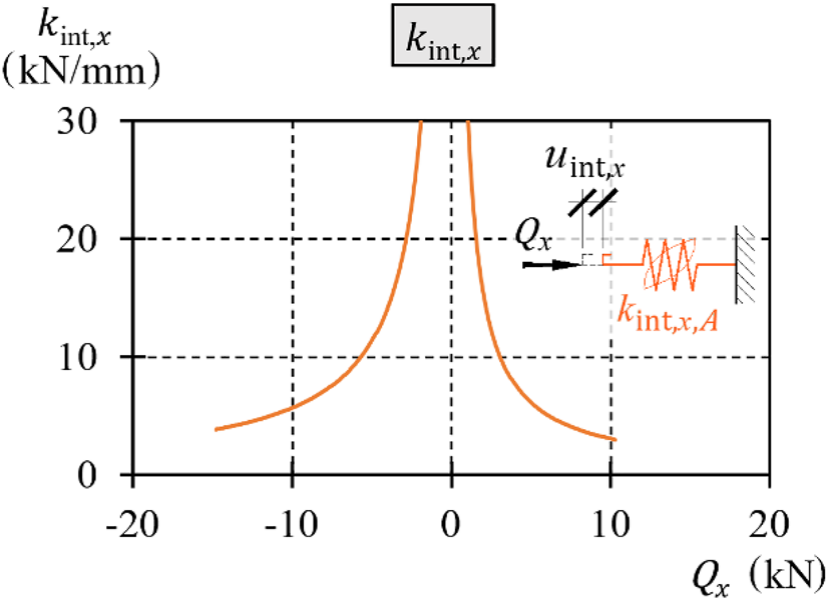
Stiffness $${k}_{{\text{int}},x,A}$$.

Functions of stiffness ${k}_{{\text{int}},x,A+}$ and ${k}_{{\text{int}},x,A-}$, which were determined on the basis of the tests and (18), are presented as graphs (Fig. 23) and in tabular form (Table 2) for selected values of the transverse load.

**Table 2:** Stiffness $${k}_{{\text{int}},x,A}$$.

Stiffness	${Q}_{x,{\text{extr}}}$ (kN)	Part of loading ${Q}_{x,{\text{extr}}}$ (%)				
		0	25	50	75	100
${k}_{{\text{int}},x,A-}$, kN/mm	− 14.8	$\infty$	15.4	7.68	5.12	3.84
${k}_{{\text{int}},x,A+}$, kN/mm	10.3	$\infty$	12.0	5.94	3.98	2.97

### Stiffness ${{\varvec{k}}}_{\mathbf{c}\mathbf{o}\mathbf{n},{\varvec{x}}}$ and parameters ${\boldsymbol{\alpha }}_{\mathbf{c}\mathbf{o}\mathbf{n}}$ and ${{\varvec{\beta}}}_{\mathbf{c}\mathbf{o}\mathbf{n}}$

The initial stiffness ${k}_{{\text{con}},x,{\text{init}}}$ was determined from a test in which only slight nonlinearity of the support was observed. Hence, it was based on the test with the smallest ${u}_{x,{\text{extr}}}$ equal to 1 mm. This stiffness, in accordance with (36), was equal to the ratio of coefficients ${\beta }_{{\text{con}}}$ to ${\alpha }_{{\text{con}}}$. Knowing the ratio of both coefficients, the least squares method was used to determine one of them for which the function (31) was the closest approximation of the path representing a difference between the relationships ${Q}_{x}-{u}_{x}$ and ${Q}_{x}{-(u}_{{\text{teor}},x}+{u}_{{\text{con}},x})$ obtained by adding up the relationships (4) and (14). The coefficients determined as specified above were ${\alpha }_{{\text{con}}-}\hspace{0.17em}$ = − 34.60, ${\alpha }_{{\text{con}}+}\hspace{0.17em}$ = 18.98, ${\beta }_{{\text{con}}-}\hspace{0.17em}$ = − 57.18 and ${\beta }_{{\text{con}}+}\hspace{0.17em}$ = − 30.82.

**Figure 24:**
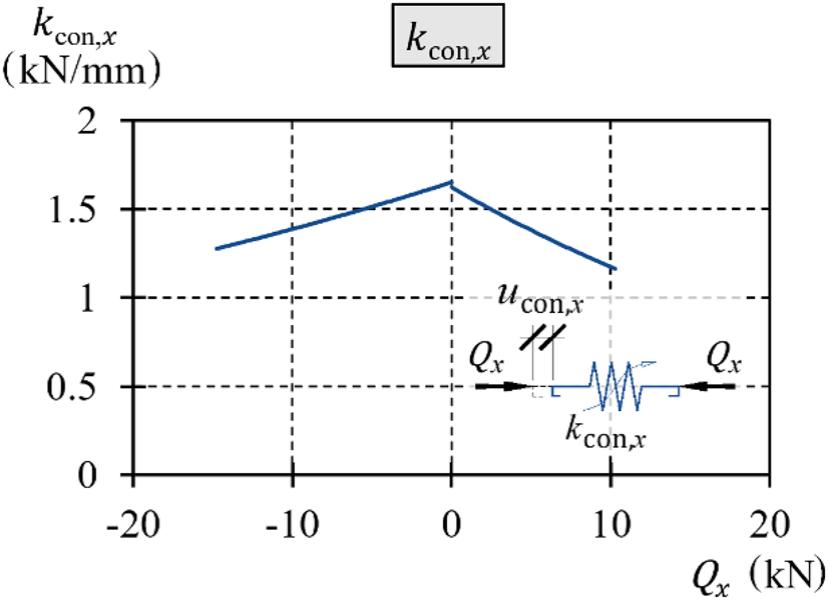
Stiffness $${k}_{{\text{con}},x}$$.

The parameters of the spring stiffness ${k}_{{\text{con}},x,-}$ and ${k}_{{\text{con}},x,+}$ determined in accordance with (35) are presented in graphical (Fig. 24) and tabular (Table 3) forms for selected values of the transverse load.

**Table 3:** Stiffness $${k}_{{\text{con}},x}$$.

Stiffness	${Q}_{x,{\text{extr}}}$ (kN)	Part of loading ${Q}_{x,{\text{extr}}}$ (%)				
		0	25	50	75	100
${k}_{{\text{con}},x-}$(kN/mm)	− 14.8	1.65	1.55	1.45	1.36	1.28
${k}_{{\text{con}},x+}$(kN/mm)	10.3	1.62	1.49	1.37	1.26	1.16

## Analysis of the model of dry stack structural elements and discussion

The defined model may be used to represent five phases in the loading‒unloading process for dry stack structural elements under a cyclic bending moment. Each phase displayed linear-elastic properties resulting from the stiffness of the idealized element’s properties, nonlinear elastic properties and friction, representing the effect of geometric imperfections. The model was characterized by four parameters, whose values were determined from test results for the supports with different configuration parameters.

**Figure 25:**
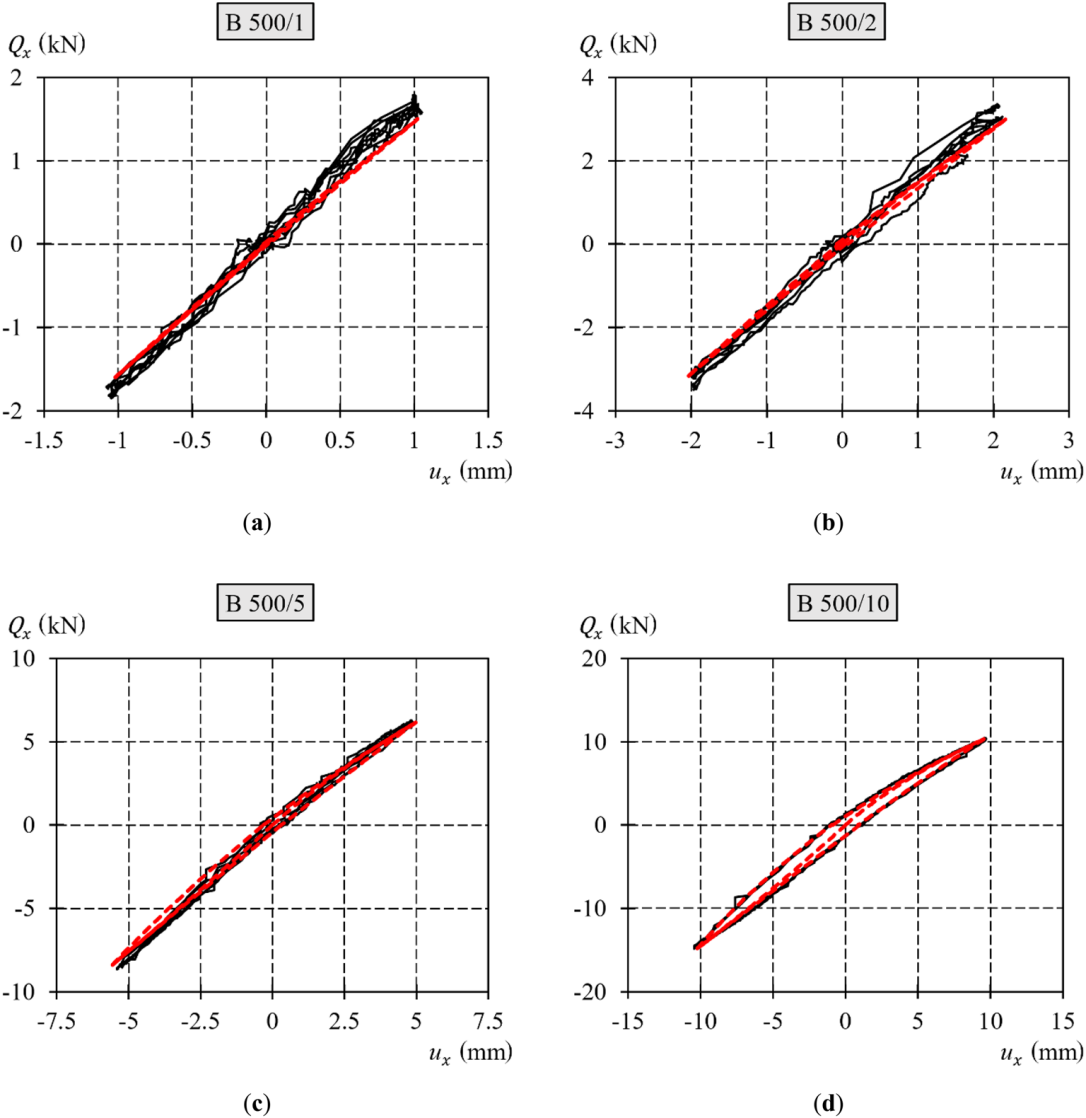
Loops of the loading‒unloading cycle with a variable sign force obtained from the tests and the model (black continuous lines are the test results; red dashed lines are the model results): (**a**) ± 1 mm; (**b**) ± 2 mm; (**c**) ± 5 mm; (**d**) ± 10 mm.

Figure 25 shows the results obtained from the model and test results. In the graphs, the lines from the model and lines from the tests practically overlap. Consequently, the assumed model was considered to be satisfactory for describing the effects observed in the supports. The relationships utilized to generate the graphs ${Q}_{x}-{u}_{x}$ are compared in Table 4.

**Table 4:** Relationships describing phases of the model.

Phase	Range of load ${Q}_{x}$	${u}_{{\text{teor}},x}$	${u}_{{\text{con}},x}$	${u}_{{\text{int}},x}$	${u}_{x}$
		According to relationship			
*A*	$0\to {Q}_{x,{\text{min}}}$	(4)	(27)	(19)	${u}_{{\text{teor}},x}\left({Q}_{x}\right)+{u}_{{\text{con}},x}\left({Q}_{x}\right)+{u}_{{\text{int}},x,A}\left({Q}_{x}\right)$
*B*	${Q}_{x,{\text{min}}}\to 0$			(19) and (22)	${u}_{{\text{teor}},x}\left({Q}_{x}\right)+{u}_{{\text{con}},x}\left({Q}_{x}\right)+{u}_{{\text{int}},x,A}\left({Q}_{x,{\text{min}}}\right)-{u}_{{\text{int}},x,B\div E}\left(\Delta {Q}_{x}\right)$
*C*	$0\to {Q}_{x,{\text{max}}}$				
*D*	${Q}_{x,{\text{max}}}\to 0$				${u}_{{\text{teor}},x}\left({Q}_{x}\right)+{u}_{{\text{con}},x}\left({Q}_{x}\right)+{u}_{{\text{int}},x,A}\left({Q}_{x,{\text{max}}}\right)-{u}_{{\text{int}},x,B\div E}\left(\Delta {Q}_{x}\right)$
*E*	$0\to {Q}_{x,{\text{min}}}$				

The preceding subsections delineated the process of determining all the parameters necessary for obtaining the transverse stiffness characteristics of the support model. The spring elements ${k}_{{\text{teor}},x}$ and ${k}_{{\text{con}},x}$ and the spring element with hysteresis ${k}_{{\text{int}},x,A}$, which form the model, were connected in series. In phase *A*, the equivalent stiffness of the elements that formed the model was named ${k}_{{\text{sup}},x,A}$ and was determined from the following relationship:

37 $${k}_{{\text{sup}},x,A}\left({Q}_{x}\right)={\left(\frac{1}{{k}_{{\text{teor}},x}\left({Q}_{x}\right)}+\frac{1}{{k}_{{\text{con}},x}\left({Q}_{x}\right)}+\frac{1}{{k}_{{\text{int}},x,A}\left({Q}_{x}\right)}\right)}^{-1}.$$

**Figure 26:**
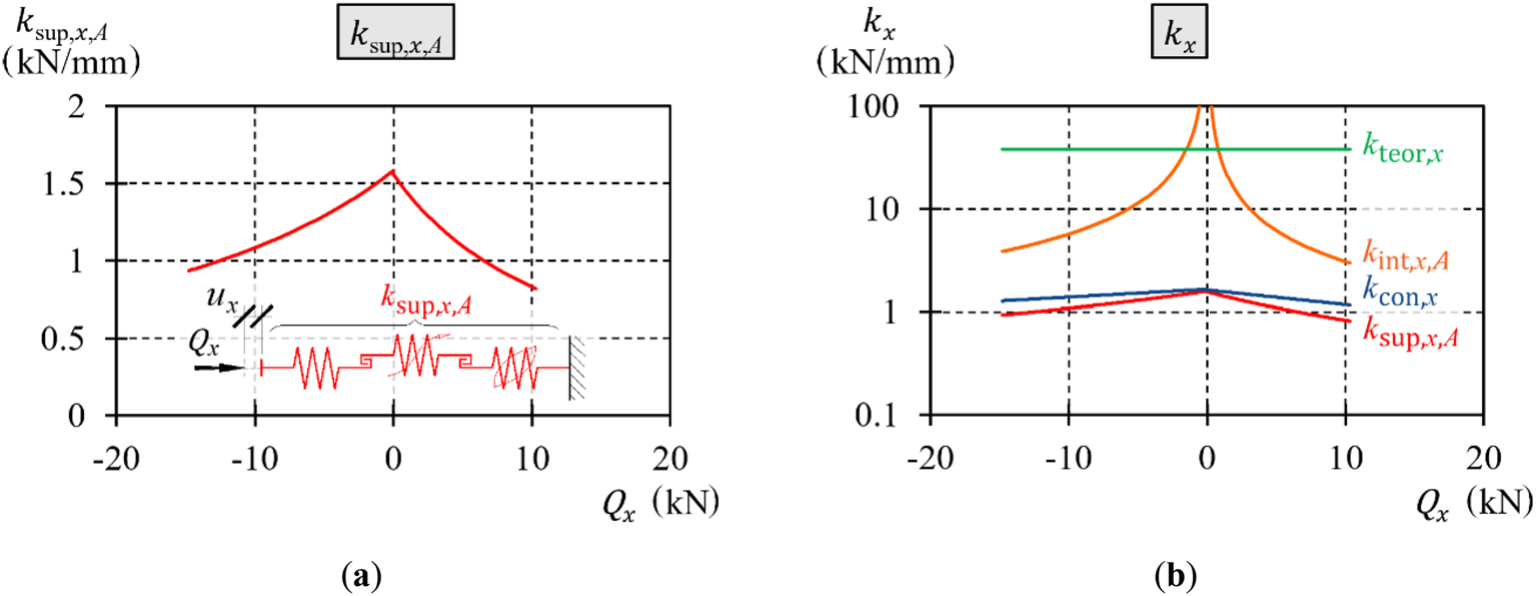
Characteristics of transverse stiffness of the support: (**a**) support; (**b**) support and its elements.

This characteristic was determined on the basis of (37) and is presented in graphical (Fig. 26) and tabular (Table 5) forms.

**Table 5:** Transverse stiffness of the support ${k}_{{\text{sup}},x,A}$.

Stiffness	${Q}_{x,{\text{extr}}}$	Part of loading ${Q}_{x,{\text{extr}}}$ (%)				
	(kN)	0	25	50	75	100
${k}_{{\text{sup}},x,A-}$(kN/mm)	− 14.8	1.58	1.36	1.18	1.05	0.934
${k}_{{\text{sup}},x,A+}$(kN/mm)	10.3	1.55	1.28	1.08	0.936	0.818

The transverse stiffness of the support decreased as the deflection and transverse load increased. This stiffness differed substantially from the theoretical transverse stiffness ${k}_{{\text{teor}},x}$. The transverse stiffnesses of the support with a longitudinal force of 500 kN did not exceed 5% of the theoretical stiffness. As the transverse load increased, the permanent displacement and the area enclosed by the hysteresis loop also increased. Additionally, the hysteresis loop was not symmetrical because the parameters of the model loaded with positive transverse force differed from the parameters of the model loaded with negative transverse force.

**Figure 27:**
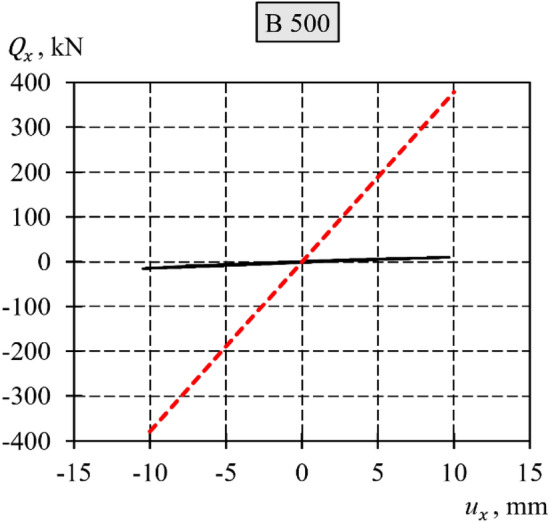
Loops of the loading‒unloading cycle with a variable sign force obtained from the tests and the spring ${k}_{{\text{teor}},x}$ (black continuous lines are the test results; red dashed lines are the model results of the spring ${k}_{{\text{teor}},x}$).

In this context, it is crucial to emphasize the influence of geometric imperfections on the support stiffness by comparing loading‒unloading cycles obtained from experimental tests with those predicted by the theoretical spring model ${k}_{{\text{teor}},x}$ (Fig. 27).

## Conclusions

Tests were conducted on an exemplary dry stack structure in the form of a temporary support composed of a stack of cuboid elements and a hydraulic jack. These tests revealed the nonlinear behaviour of the support when subjected to cyclic bending moments. The transverse stiffness of the support decreased as both the deflection and transverse load increased. Furthermore, hysteresis loops were observed during the cyclic bending moment.

The authors proposed a mathematical model of dry stack structural elements subjected to a cyclic bending moment. This model captured the physical phenomena of microslips between the elements of the stack and closing gaps, which results in the nonlinear behaviour of such systems. The model exhibited a commendable level of concordance between the calculated outcomes and experimental findings.

Despite the complexity of the mechanical behaviour of dry stacks, the authors have successfully developed a relatively simple model. This model consists of only three elements defined by four parameters. The first element represents a linear spring that corresponds to the mechanical behaviour of the idealized structural element without any imperfections, exhibiting linear characteristics. The other two elements (nonlinear springand spring with hysteresis) correspond to the effects of geometric imperfections and the presence of dry joints, resulting in a reduction in stiffness and the occurrence of friction forces, leading to the formation of a hysteresis loop during cyclic loading.

The ability of the algorithm to determine model parameters based on cyclic load tests was verified through the analysis of a temporary support consisting of a stack of cuboid elements and a hydraulic jack. Furthermore, the model demonstrated strong concordance between the calculated results and experimental observations. The described model of dry stack structural elements can be applied to other structures that demonstrate similar phenomena, leading to similar hysteresis loops and nonlinearities. This could include, for instance, structural joints with friction.

The proposed model can be classified as a semiempirical model and can be considered a starting point for conducting further analyses, such as parametric analyses, which can help predict the mechanical behaviour of a system in different scenarios. It can also assist in determining dynamic parameters, such as the lowest frequency of free vibrations and the damping of dry stack structures.

## Data Availability

All the data generated or analysed during this study are included in this published article.
